# Deep Learning-Based Pan-Cancer Classification Model Reveals Tissue-of-Origin Specific Gene Expression Signatures

**DOI:** 10.3390/cancers14051185

**Published:** 2022-02-24

**Authors:** Mayur Divate, Aayush Tyagi, Derek J. Richard, Prathosh A. Prasad, Harsha Gowda, Shivashankar H. Nagaraj

**Affiliations:** 1 Centre for Genomics and Personalised Health, Queensland University of Technology, Brisbane, QLD 4059, Australia; mayurdashrath.divate@hdr.qut.edu.au (M.D.); derek.richard@qut.edu.au (D.J.R.); 2 Indian Institute of Technology, IIT Delhi Main Rd., IIT Campus, Hauz Khas, New Delhi 110016, India; aayush16081@iiitd.ac.in (A.T.); prathoshap@ee.iitd.ac.in (P.A.P.); 3 Translational Research Institute, 37 Kent Street, Brisbane, QLD 4102, Australia; 4 Department of Electrical Communication Engineering, Indian Institute of Science, Devasandra Layout, Bengaluru 560012, India; 5 QIMR Berghofer Medical Research Institute, 300 Herston Rd., Brisbane, QLD 4006, Australia; 6 Faculty of Health, Queensland University of Technology, Brisbane, QLD 4059, Australia; 7 Faculty of Medicine, The University of Queensland Mayne Medical School, 20 Weightman Street, Brisbane, QLD 4006, Australia

**Keywords:** deep learning, pan cancer, cancer type prediction, gene expression signatures

## Abstract

**Simple Summary:**

Gene expression data from different cancer types offer the opportunity to identify cancer tissue-of-origin specific biomarkers and targets. In this study, we used pan-cancer gene expression data to train a deep learning neural network model to identify cancer tissue-of-origin specific gene expression signatures. We identified 976 genes that can reliably classify different cancer types with >97% accuracy.

**Abstract:**

Cancer tissue-of-origin specific biomarkers are needed for effective diagnosis, monitoring, and treatment of cancers. In this study, we analyzed transcriptomics data from 37 cancer types provided by The Cancer Genome Atlas (TCGA) to identify cancer tissue-of-origin specific gene expression signatures. We developed a deep neural network model to classify cancers based on gene expression data. The model achieved a predictive accuracy of >97% across cancer types indicating the presence of distinct cancer tissue-of-origin specific gene expression signatures. We interpreted the model using Shapley additive explanations to identify specific gene signatures that significantly contributed to cancer-type classification. We evaluated the model and the validity of gene signatures using an independent test data set from the International Cancer Genome Consortium. In conclusion, we present a robust neural network model for accurate classification of cancers based on gene expression data and also provide a list of gene signatures that are valuable for developing biomarker panels for determining cancer tissue-of-origin. These gene signatures serve as valuable biomarkers for determining tissue-of-origin for cancers of unknown primary.

## 1. Introduction

The identities and phenotypes of different cell types are governed by underlying gene expression pattern. While all cells contain the same genetic information, only a subset of genes are expressed in a given cell type. Identification of unique gene expression signatures associated with different cancers are valuable as diagnostic biomarkers and therapeutic targets. For example, the expression of prostate-specific antigen (PSA) is elevated in prostate cancer patients [1]. Identification of such signatures requires pan-cancer studies that investigate gene expression pattern associated with different cancer types. However, until recently, a large number of gene expression profiles from multiple cancer types were not available. The advent of high-throughput sequencing methods has revolutionized the field of cancer genomics and transcriptomics studies. The Cancer Genome Atlas (TCGA) and International Cancer Genome Consortium (ICGC) have sequenced hundreds of samples from multiple cancer types and made this data available to the scientific community [2,3].

Advent of computational method called deep learning has enabled researchers to utilize multilayer neural networks to analyze these large data sets. Deep learning is a cutting-edge artificial intelligence technique that provides a powerful framework for supervised learning [4]. According to the universal approximation theorem [5,6], a feedforward network with linear output and at least one hidden layer with any squashing activation function can approximate any decision boundary to arbitrary closeness if provided with sufficient hidden units. Importantly, deep learning has already been shown to achieve higher accuracy and superior performance relative to traditional machine learning (ML) techniques including support vector machines and random forest approaches [4]. Researchers have applied deep learning to predict cancer types based on various omics data such as mutation profile [7,8,9] and gene expression data [10,11,12]. In addition, previous studies attempted to developed deep learning models utilizing multi-omics data to predict cancer type [13] and cancer patient survival [14,15]. However, one major limitation of using multi-omics data is the lack of large multi-omics pan-cancer data sets. Often, two or more types of omics data for the same sample are not available. Machine learning and deep learning have also been utilized for cancer type classification and gene signature identification [12,16]. Furthermore, in addition to the availability of model interpretation methods, researchers have also developed interpretable deep learning frameworks. For example, interpretable deep learning model architectures, such as a capsule network, have been used to identify gene signatures associated with cell subtypes in single-cell RNA-seq data [17]. Another example is the GenNet interpretable deep learning framework, which was used to uncover genes linked to various traits [18].

We carried out a comprehensive pan-cancer gene expression analysis using deep learning to analyze transcriptome data from TCGA to identify cancer tissue-of-origin specific gene signatures. We developed a model that classified different cancer types based on transcriptomic data with >97% accuracy. Interpretation of this model revealed 976 genes that constitute cancer tissue-of-origin specific gene expression signatures including specific genes that can effectively discriminate among different cancer types. These gene signatures can be used to guide the development of more reliable gene panels for tissue-of-origin diagnosis and monitoring of cancers. In addition, this DNN model can be extended to identify tissue-of-origin for cancers of unknown primary.

## 2. Materials and Methods

### 2.1. Gene Expression Data Collection and Pre-Processing

We obtained fragment per kilobase per million mapped fragments (FPKMs) values for 14,237 tumor samples representing 39 cancer types from the TCGA data portal (https://portal.gdc.cancer.gov, accessed on 20 August 2020) using the GDC data transfer tool. We analyzed only protein-coding genes. In order to reduce noise and to focus on genes that were reliably expressed, we excluded genes that did not exhibit expression levels ≥ 5 FPKM in at least 50% of samples in a given cancer type. These gene lists were then merged to create a non-redundant gene list for model construction. FPKM values were log10 transformed before using the data for model training and testing. Sample labels (i.e., cancer types) were encoded using OneHotEncoder from the sklearn python package. Samples of each cancer type were randomly assigned to three data sets: (1) a training set (70%), (2) a validation set (10%), and (3) a testing set (20%).

### 2.2. Unsupervised Clustering

T-distributed stochastic neighbor embedding (t-SNE) is an unsupervised nonlinear dimensionality reduction technique [19]. We employed t-SNE before and after log-transformation of data for data visualization. For this, the t-SNE method from the sklearn package was used for projecting data into 2 dimensions by setting the random number initialization to 123 and the rest of the parameters at default values.

### 2.3. Implementation Details

We used a deep neural network (DNN) for developing, testing, and validating the model for the classification of cancer types based on gene expression data. The model architecture and training were implemented on an NVIDIA Tesla M60 GPU with 128 GB RAM using TensorFlow 2.0 and Python 3 [20]. The DNN consisted of five fully connected (FC) hidden layers with 500, 250, 125, 100, and 75 nodes. The output layer had 37 nodes, each representing one of the cancer types. The rectified linear unit (ReLU) activation function was used for all hidden layers, while SoftMax was used for the output layer. A grid search was performed to identify the best optimization algorithm and learning rate using TesnsorBoard HParams [20]. For this strategy, we used the Adam [21], Stochastic gradient descent [22], and RMSprop [23] optimizers with learning rates of 0.001, 0.0001 and 0.00001. The model was trained with a learning rate of 0.00001 and Adam optimizer [21] that were selected during grid search. As it was a multiclass classification problem, we used categorical cross-entropy as a loss function and one-hot encoded labels. In order to avoid model overfitting, we used early stopping to monitor the loss function with patience equal to 3 and with the maximum number of permitted epochs being 50.

$$Categorical\;cross-entropy=-\overset{n}{\underset{i=1}{\sum }}p\left(x_{i}\right)\;\mathrm{Log}\left(q\left(x_{i}\right)\right)$$

where *n* is the total number cancer types, *p(x_i_)* is the probability of cancer type *x_i_*, and *q(x_i_)* is the predicted probability of cancer type *x_i_*.

### 2.4. Evaluation of the Model’s Performance

The model’s performance was assessed using the most commonly used evaluative metric, i.e., accuracy. However, it tends to be biased towards over-represented classes for class imbalanced data. As such, cases existed in our data set; thus, we also used precision, recall, and F1-score values to assess model performance on the test data.

### 2.5. Model Interpretation Based upon SHAP Values

To identify the most important model driving genes, we conducted model interpretation using Shapley additive explanations (SHAP) values [24]. SHAP is a method that scores each feature according to its contribution to the model’s prediction. A feature with the highest SHAP value is the major contributor to the output of a model [24]. We built 10 different DNN models using randomly split TCGA data, and SHAP values for each of those models were computed using the DeepExplainer function from the Python SHAP package. The top 20 genes were selected for each cancer type in every model based on absolute mean SHAP values. Then, these genes were compared across cancer types based on their median FPKM values to establish expression signatures.

### 2.6. ICGC Test Data Set

To test the model on an independent data set apart from TCGA, we downloaded gene expression data pertaining to the latest release from the ICGC data portal (https://dcc.icgc.org/releases/current/Projects, accessed on 26 August 2021). We first removed samples that were common between ICGC and TCGA data sets. We also discarded normal tissue samples. Additionally, we eliminated Ewing’s sarcoma (BOCA-FR) and pediatric brain tumor (PBCA-US) cohorts. As the model was not trained using Ewing’s sarcoma and PBCA-US data with transcript-level expression. In total, there were 2085 samples spanning across 18 cancer types in the resultant data set. ID mapping was performed using ensemble BioMart and HGNC Multi-symbol checker tools. Read count was set to zero for missing genes. Before using the data for model testing, the FPKM values were calculated and log10 transformed.

### 2.7. Implementation Details of Cloud-Based Web Tool

The backend was built using Amazon Web Services (AWS), while the user interface was designed using Angular framework, CSS, and HTM5. Simple Storage Service (S3) buckets were used to store input and output files. A docker image was created to analyse the input data. It was configured to support the scikit-learn and TensorFlow2 framework using python 3.7. Elastic Container Repository (ECR) was used to deploy the docker image. A lambda function was created to communicate between the S3 bucket and ECR docker image. The frontend and backend were linked using the REST API.

## 3. Results

### 3.1. Unique Gene Expression Signatures Are Associated with Different Cancer Types

We used transcriptome data from 14,237 tumor samples from 39 cancer types available on the TCGA data portal, including adenocarcinomas, squamous cell carcinomas, haematological malignancies, neuronal tumors, melanomas, germ cell tumors, soft tissue tumors, and other cancer types (Supplementary Table S1). The data was class imbalanced. For example, there are 1176 samples from breast invasive carcinoma (BRCA) while there are only 34 samples from cholangiocarcinoma (CHOL) (Supplementary Table S1). We combined proximal cancers, as they show high molecular similarity. For example, colon and rectal cancer data were combined and labelled as colorectal cancer (CORE). Similarly, uterine carcinosarcoma (UCS) was combined with uterine corpus endometrial carcinoma (UCEC). As a result, the data used for this analysis comprised 37 cancer types (Supplementary Table S1).

**Figure 1:**
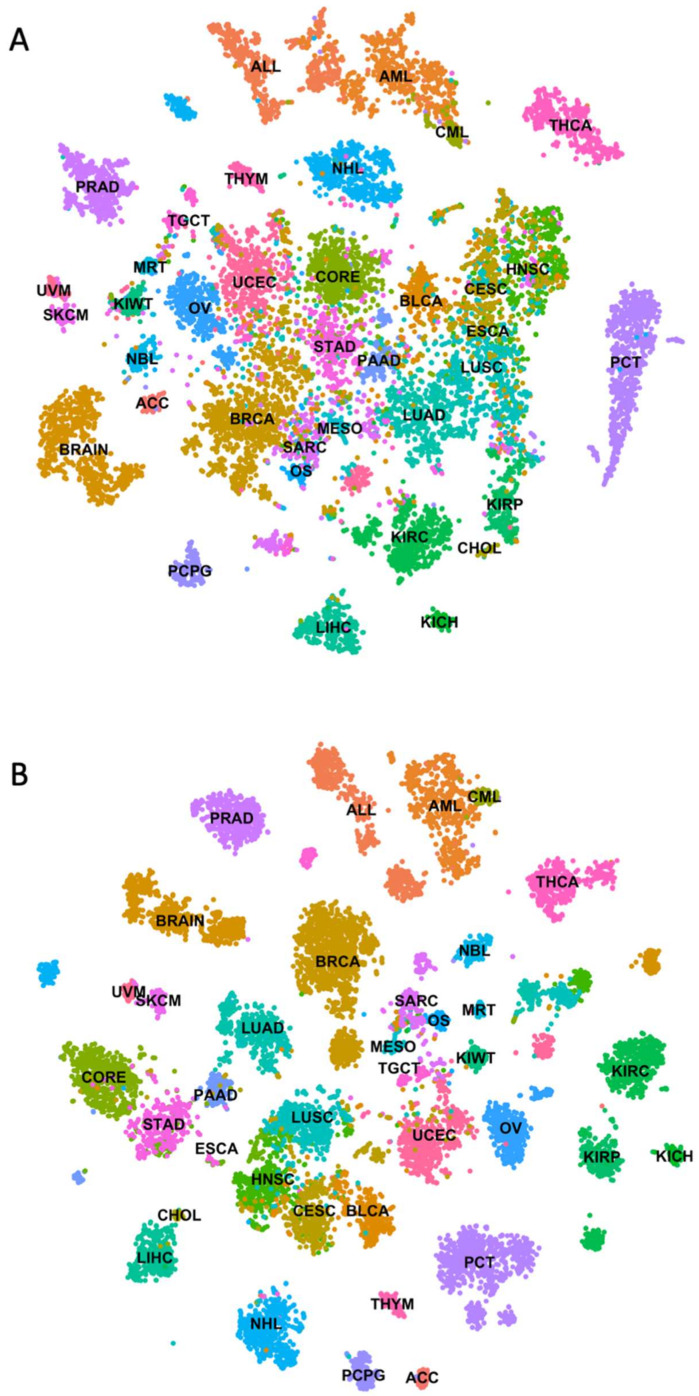
Unsupervised clustering of the cancer genome atlas RNA-seq data using t-distributed stochastic neighbor embedding. (**A**) t-distributed stochastic neighbor embedding plot showing unsupervised clustering of transcriptome data from the cancer genome atlas without log transformation (**B**) t-distributed stochastic neighbor embedding plot showing unsupervised clustering of transcriptome data from the cancer genome atlas after log transformation.

RNA-seq is one of the most powerful techniques for gene expression profiling. However, accurate noise-free quantification remains a challenge [25]. Deep learning models are sensitive to noise in underlying data. Genes that are expressed at low levels represent a potential source of noise as their measurement across samples can be inconsistent. To eliminate such noise and consider only genes that are reliably expressed in tissues of interest, we filtered data to only include genes with ≥5 FPKM in at least 50% of samples within a cancer type. This reduced the total number of genes from 19,801 to 13,250. As recommended for the skewed data distribution, FPKM values were log-transformed before using the data for model building [26]. As can be seen from Figure 1, it reduced data variance significantly (Supplementary Table S2).

When log-transformed data was used for the t-SNE unsupervised clustering, it exhibited better separation between cancer types (Figure 1B). As expected, cancer types originating from the same tissues or cell types formed overlapping or proximal clusters due to similar underlying gene expression profiles, such as skin cutaneous melanoma (SKCM) and uveal melanoma (UVM), acute myeloid leukemia (AML) and chronic myeloid leukemia (CML), colon and rectal (CORE), and uterine carcinosarcoma and uterine corpus endometrial carcinoma (UCEC) (Figure 1B). Liver hepatocellular carcinoma (LIHC) and CHOL were also clustered near one another owing to their proximity. Interestingly, cancers originating from squamous cells of different tissues such as lung squamous cell carcinoma (LUSC), head and neck squamous cell carcinoma (HNSC), cervical squamous cell carcinoma and endocervical adenocarcinoma (CESC), and bladder urothelial carcinoma (BLCA) also clustered proximally compared to adenocarcinomas derived from the same tissue types. Esophageal carcinoma (ESCA) samples, which included both adenocarcinoma and squamous cell carcinoma tissues, formed overlapping clusters with stomach adenocarcinoma (STAD) and squamous cell cancers. These clustering patterns reveal that unique gene expression profiles are associated with different cancer types.

### 3.2. Development and Training of a Deep Neural Network Model

**Figure 2:**
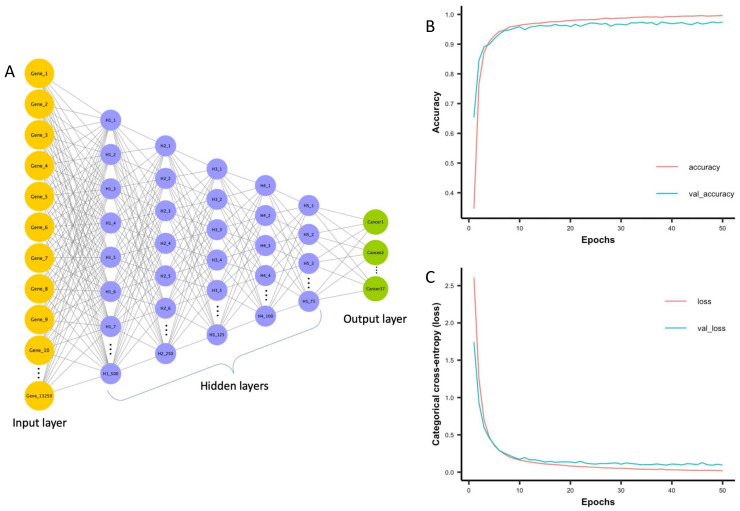
Architecture of deep neural network model and its progress during training. (**A**) The deep neural network model consists of an input layer, five hidden layers and an output layer. The input layer takes input fragment per kilobase per million mapped fragments values for 13,250 genes. Hidden layers perform nonlinear transformation using rectified linear unit activation function to distinguish between cancer types. There are 5 hidden layers with 500, 250, 125, 100 and 75 nodes. The output layer consists of 37 nodes each representing one of the cancer types. (**B**) The accuracy and (**C**) Categorical cross-entropy (loss) of deep neural network model on both training and validation data sets.

We developed a pan-cancer classification model using deep neural networks (DNN) and RNA-seq data from TCGA. We opted for DNN based architecture as it is better suited to learning latent patterns in data where input variables are independent of one another. Our first DNN model was composed of 5 dense hidden layers with 50 nodes, and its accuracy was below 40%. The learning capacity of the model was improved by adjusting the number of hidden layers and nodes which resulted in improved model accuracy of over 95%. Then we performed a grid search to identify the best optimization algorithm and learning rate using TensorBoard HParams [20]. Our final DNN model consisted of 5 hidden layers with 500, 250, 125, 100, and 75 nodes, respectively (Figure 2A). It was trained using Adam optimization algorithm to minimize the categorical cross-entropy loss function, and the learning rate was set to 0.00001. It took 103 seconds to train the model. Figure 2B shows the accuracy and loss function during the training process of the model. The DNN model took only 5 epochs to reach more than 90% accuracy and loss (categorical cross-entropy) as low as 0.28 without exhibiting obvious overfitting. During training, two data splits were used: one for training (70%) and another for validation (10%). The model was repeatedly tested on validation data at the time of training to monitor its performance and to prevent any overfitting. The overall training accuracy of the model was 99.64%, and the validation accuracy was 97.40%. To examine the consistency of our model architecture, we randomly split data 10 times into training, validation, and test data sets. We obtained consistent results (Supplementary Figure S1).

### 3.3. DNN Model Accurately Predicts Cancer Types Based on Gene Expression Profiles

**Figure 3:**
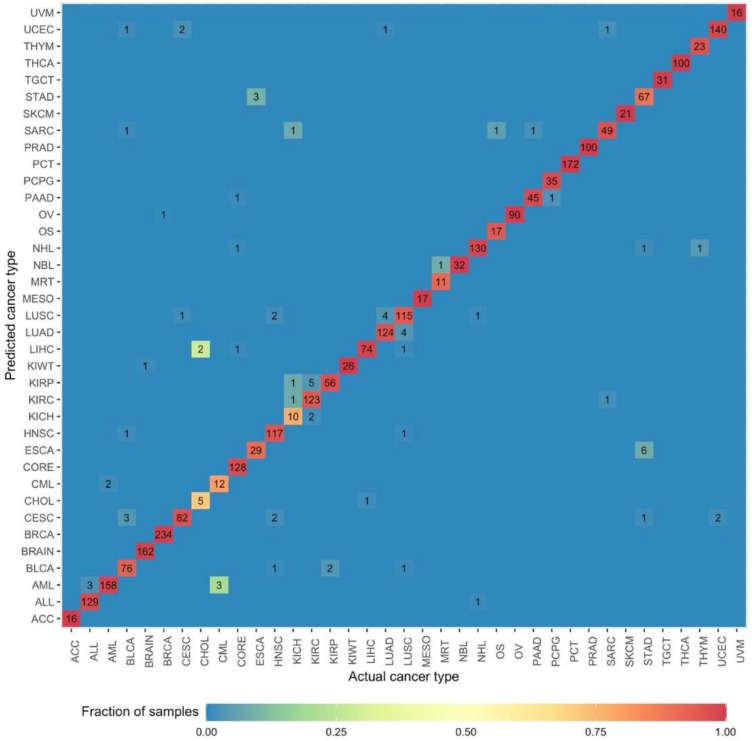
Performance of DNN model on the test data set.

To evaluate the performance of DNN model, we used it to classify a test data set containing gene expression data from 2848 samples corresponding to 37 different cancer types. The model accurately predicted cancer type for 2772 tumor samples and misclassified 76 samples (Figure 3). The overall model accuracy on test data was 97.33. The weighted averages for precision, recall, and f1-score values were 97.37%, 97.33%, and 97.33%, respectively. For most cancers, the test accuracy was above 95%. The test accuracy was 100% for the following 11 cancer types: adrenocortical carcinoma (ACC), kidney Wilms’ tumor (KIWT), mesothelioma (MESO), neuroblastoma (NBL), ovarian serous cystadenocarcinoma (OV), plasma cell tumors (PCT), prostate adenocarcinoma (PRAD), testicular germ cell tumors (TGCT), thyroid carcinoma (THCA), SKCM, and UVM. The test accuracy for kidney renal clear cell carcinoma (KIRC), malignant rhabdoid tumors (MRT), osteosarcoma (OS), BLCA, ESCA, and LUSC were between 90% to 95%. The test accuracy was below 90% for four cancer types: kidney chromophobe (KICH), CHOL, CML and STAD.

Actual cancer type is represented on the *x*-axis and predicted cancer type is represented on the *y*-axis. Number of samples in the test data set that were accurately classified are represented on the diagonal.

The model performed poorly when classifying CHOL samples, with a test accuracy of 71.42%. This may be due to the limited number of samples in the training data set and the anatomical location of this cancer type. Small sample sizes hinder model learning and result in the construction of a poor classifier. CHOL originates from the liver and performing a biopsy of these tumors without liver cell contamination is non-trivial. Hence, CHOL samples often exhibit gene expression profiles similar to those associated with LIHC. This was also evident from t-SNE unsupervised clustering results where these two cancer types formed proximal clusters (Figure 1B). Even though only 2 (out of 7) samples were misclassified, this significantly reduced test accuracy for CHOL. Interestingly, both misclassified samples were predicted as LIHC samples.

Classification ambiguities were also observed among different cancers arising from the same organ systems. For example, three acute lymphoid leukemia (ALL) samples were predicted as AML samples, and four lung adenocarcinoma (LUAD) samples as LUSC. Same was observed for kidney renal papillary cell carcinoma (KIRP), KIRC, KICH, CESC, UCEC, STAD, and ESCA samples (Figure 3). Classification ambiguity among cancers originating from proximal tissues also highlights the challenges associated with their diagnosis. For example, biopsy sites for 70% of ESCA samples were in the lower third of the esophagus closer to the gastroesophageal junction. The precision score was lowest for ESCA (82.85%), as six STAD samples were predicted as being ESCA samples. CHOL cancer exhibited the lowest recall (sensitivity) score and f1-score values at 71.42% and 76.92%, respectively.

We performed stratified five-fold cross-validation of our model by randomly dividing TCGA data into five data sets. Each time, we used one of those data sets as a test set and the remaining four as a training set for the model. Our model achieved consistent results in this cross-validation analysis, with a training accuracy of more than 99% (Supplementary Table S3). Precision, recall, and f1-scores were also consistent. Overall, the model predicted different cancer types with over 97% accuracy.

### 3.4. Identification of Cancer Tissue-of-Origin Specific Gene Expression Signatures

**Figure 4:**
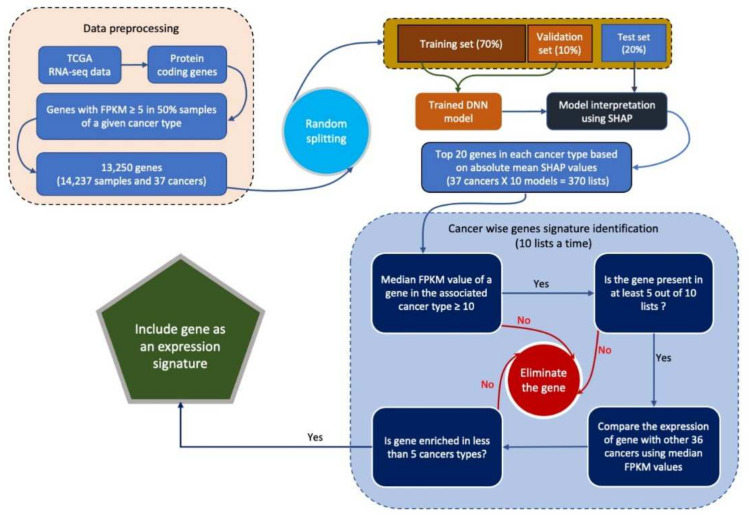
Workflow employed to identify gene expression signatures. A schematic showing workflow employed to identify gene expression signatures using deep learning model and SHAP values.

**Figure 5:**
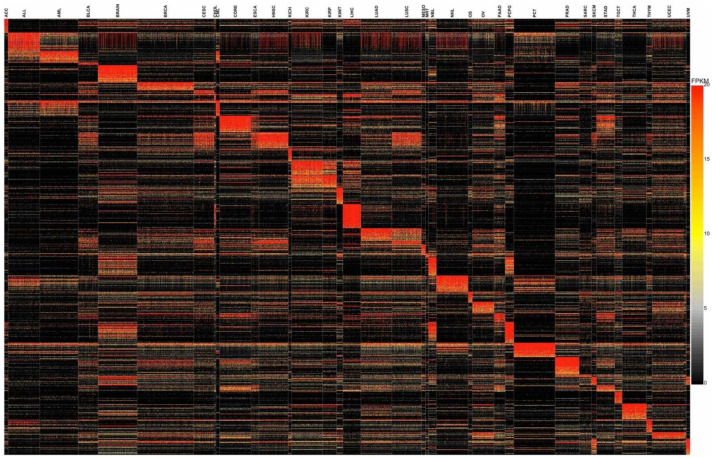
Cancer tissue-of-origin specific gene signatures. Supervised hierarchical clustering to show gene expression signatures associated with different cancer types.

**Figure 6:**
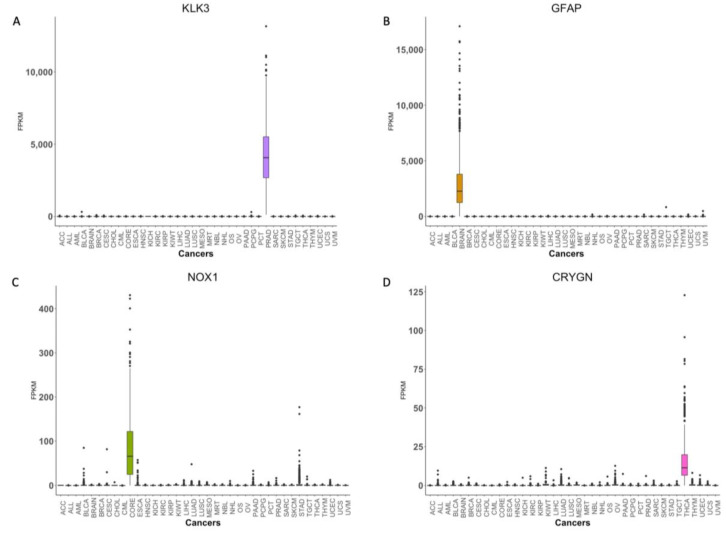
Representative examples of genes that exhibit cancer tissue-of-origin specific expression. Box-Whisker plots showing expression of (**A**) KLK3, (**B**) GFAP, (**C**) NOX1 and (**D**) CRYGN across 37 cancer types in TCGA data set.

We next used SHAP values to interpret our model to identify gene signatures that drive classification [24]. To identify gene signatures associated with each cancer type, we developed 10 DNN models with the same architecture. Figure 4 shows the workflow employed to identify gene expression signatures. To ensure that non-identical data was used during the construction of each model, the TCGA data set was randomly divided into training (70%), validation (10%), and test (20%) data sets. The performance of all 10 models was consistent and they all achieved 99% and 97% accuracy during training and testing, respectively (Supplementary Figure S1). Next, we calculated SHAP values for each gene using the test data set for the respective model. In each model, the top 20 genes were selected for each cancer type based on absolute mean SHAP values. Then, we calculated the occurrence frequency—number of times a gene was among the top 20 genes associated with a particular cancer type. Only genes with an occurrence frequency of at least 5 for a given cancer type were selected. This resulted in the selection of 507 candidate genes. Only genes that were enriched in less than five cancer types were retained. This resulted in the identification of 448 gene expression signatures. We repeated the above procedure to identify additional gene expression signatures after excluding these 448 genes. During the second iteration, we identified 310 genes and an additional 218 genes in the third iteration. We stopped after the third iteration as we were unable to detect any gene signature for BLCA, ESCA, MESO, or sarcoma (SARC). In total, we identified 976 gene signatures across 37 cancer types (Figure 5 and Supplementary Table S4). There were 55 genes that exhibited liver cancer tissue-of-origin specific expression pattern. Signatures with an occurrence frequency of 10 that were enriched in only one cancer type can be considered highly specific biomarkers. There were 134 such genes associated with 27 different cancer types in the current study (Supplementary Table S4). These 134 genes included well-known cancer biomarkers such as KLK3, which is specific to prostate cancer. Other examples included GFAP in brain cancer (BRAIN), CRYGN in THCA, and NOX1 in CORE (Figure 6). Identification of these cancer tissue-of-origin specific gene signatures demonstrates the validity of our approach.

To evaluate the validity of gene signatures identified through our model interpretation, we built a new DNN model using expression profiles of only these 976 gene signatures. The model architecture remained the same except for the input layer, which was reduced from 13,250 to 976. The model exhibited consistent performance comparable to the original model and achieved 99% and 97% training and testing accuracy, respectively. Similarly, a weighted average of precision, recall, and f1-score values were consistent with the original model (97%). The model achieved consistent performance even during five-fold cross-validation (Supplementary Figure S2). We performed unsupervised t-SNE clustering of complete TCGA data by supplying expression values corresponding to 976 gene signatures. It produced distinct clusters for the majority of cancers (Supplementary Figure S3). As expected, anatomically related cancers formed proximal clusters. The melanoma subtypes SKCM and UVM formed adjacent but well-separated clusters. These results demonstrate that our DNN model captures cancer tissue-of-origin specific gene expression signatures through enhanced pattern recognition.

### 3.5. Gene Signature Validation Using an Independent Test Data Set

To validate the findings on an independent data set, we used ICGC data. The ICGC test data was composed of primary and metastatic tumor samples from 18 cancer types including AML, BRAIN, BRCA, CESC, CORE, HNSC, KIRC, KIRP, LIHC, LUAD, neuroblastoma (NHL), OV, pancreatic adenocarcinoma (PAAD), PRAD, SKCM, STAD, THCA and UECE. The DNN model correctly predicted cancer types for 1905 out of 2085 samples. The overall accuracy of the model was 91.37%. Interestingly, our model predicted the correct cancer type for 348 of 395 metastatic samples (88.10%) and classified the following eight cancers with 100% accuracy: AML, BRAIN, HNSC, KIRP, LUAD, PRAD, THCA and UCEC. The model accuracy was below 90% for just four cancers, which included BRCA (89.21%), CORE (80%), PAAD (73.84%), and CESC (33.33%). Most of the misclassified PAAD samples were derived from the PACA-CA cohort (74/102), of which 15 and 59 samples had 14 and 35 missing gene signatures, respectively.

### 3.6. Expression Profile of Keratins across Cancer Tissues

Keratin staining is widely used in cancer diagnosis. Most epithelial cells express keratins. The human genome encodes 54 different keratins, some of which exhibit a cell/tissue-specific expression pattern [27]. Owing to their diverse expression pattern, keratins are widely used in the histopathological evaluation of cancers [28]. For example, keratins KRT8, KRT18, and KRT19 are expressed in most adenocarcinomas [27]. Ovarian cancer cells are KRT7^+^/KRT20^−^, whereas prostate cancer cells are negative for both KRT7 and KRT20 [29,30]. Many of these keratins were not identified as cancer tissue-of-origin specific gene signatures by our model. In order to evaluate, we compared keratin expression across cancer types where these markers are widely used. Surprisingly, our findings contradicted some published observations. For example, previous studies have reported that KRT8, KRT18, and KRT19 are expressed in most cancers, including skin cancer [28], whereas our analysis revealed that their expression is low in skin cancer samples (Supplementary Materials Figure S4). Similarly, KRT5, KRT6, KRT14, and KRT17 are known to be expressed by both breast cancer and SKCM. This was inconsistent with the expression pattern observed in the TCGA data set. These keratins exhibited significantly lower expression levels in almost half of the SKCM samples (Supplementary Materials Figure S4). Interestingly, KRT10, which is considered to be SKCM-specific keratin, was detected in all cancers including brain and blood cancers. Despite their widespread use in histopathology, our data suggest that there may be better alternatives to keratins that show tissue specificity. As such, our pan-cancer gene expression signature may be valuable for the development of novel immunohistochemical markers for cancer tissue-of-origin diagnosis.

### 3.7. Web Tool for Cancer-Type Prediction

We developed a web portal called Deep Learning Model for Cancer Type Prediction (Deepcap, www.deepcap.org, accessed on 26 August 2021). It allows users to upload gene expression data and obtain cancer classification. AWS cloud services, such as simple storage system (S3), elastic container repository (ECR), and lambda, were used to implement a serverless architecture (Supplementary Materials Figure S5). The S3 buckets were used to store input and output files. While ECR was used to deploy a docker image that contained the deep learning model and Python code to process the input data. Deepcap accepts gene expression matrix of 976 gene signatures as input in a comma-separated value (CSV) format. The sample names must be in the first column, and the gene symbols must be in the first row of the input file. Gene expression values must be log10 transformed FPKM values with pseudo-count one. We created a lambda function to communicate between the S3 bucket and ECR docker image. When a user uploads an input file, it automatically triggers the lambda function. The data analysis is then initiated by sending the input file’s S3 location to the docker image.

**Figure 7:**
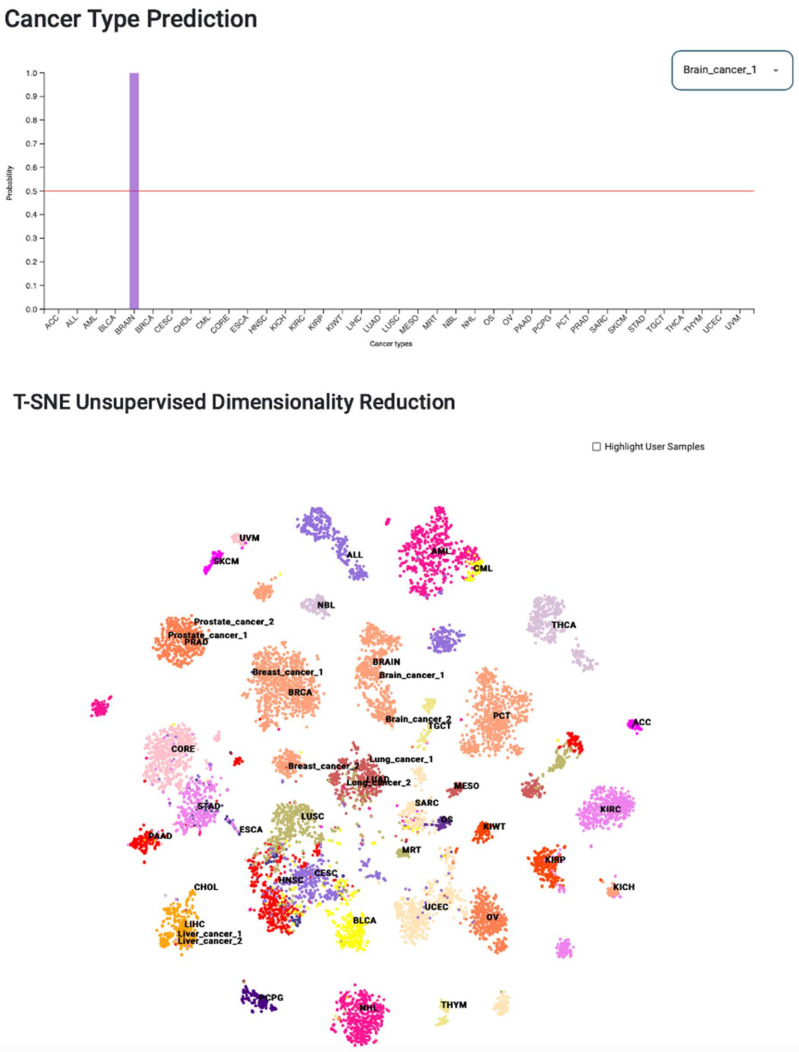
Deepcap webtool for cancer tissue-of-origin prediction and visualizing results.

Deepcap generates two types of results: (1) cancer-type predictions and (2) t-SNE analysis. For a given sample, the DNN model assigns a probability value to each of the 37 cancer types. The cancer type of the input sample is the one with the highest probability value. Deepcap provides a bar chart to compare probability values (Figure 7). A dropdown menu is provided to select the results of the sample of interest. Deepcap also performs unsupervised nonlinear dimensionality reduction using t-SNE. It enables users to visually compare their data with the training data of the model (TCGA data). A scatter plot is generated using principal components 1 and 2 to visualize the t-SNE results. The “highlight user samples” option allows the user to view only their own data on the scatter plot. Deepcap results can be downloaded by clicking on the download button.

The bar chart shows cancer-type probabilities predicted by the DNN model prediction for a given sample, while the scatter plot represents a 2D visualization of the user’s data with respect to TCGA data.

## 4. Discussion

One of the primary goals of cancer genomics and transcriptomics studies is identification of cancer biomarkers and therapeutic targets. Availability of cancer genome and transcriptome sequencing data from multiple cancer types has enabled pan-cancer analyses to identify cancer tissue-of-origin specific biomarkers and therapeutic targets. In this study, we utilized transcriptomic data from 14,237 samples from the TCGA database to carry out pan-cancer analysis and identified gene expression signatures associated with different cancer types. We used a deep learning approach for pan-cancer analysis and identified cancer-specific gene expression signatures. Our model was capable of predicting cancer types with greater than 97% accuracy based on gene expression data alone. Classification ambiguities were primarily observed when comparing different cancer subtypes within a given organ system such as tumors of the blood, kidney, lung, and gastrointestinal organs. This is likely attributable to the high degree of molecular similarity between different cancer types originating from the same tissues. In this study, we successfully implemented the SHAP method and identified a comprehensive list consisting of 976 pan-cancer gene expression signatures. These gene signatures were evaluated for their ability to accurately identify different cancer types. The model showed consistent performance, achieving over 97% accuracy on an independent test data set. Statistical measures that are not biased towards over-represented cancer types, including precision, recall, and f1-score values, were also above 97%, and the five-fold cross-validation of our model yielded consistent results. Furthermore, to assess the generalizability of the model, we used ICGC data as an independent test data set from TCGA. The model classified ICGC data with a 91.37% accuracy. The ICGC data set consisted of 395 metastatic tumor samples of which 348 were correctly classified. In other words, our model was capable of predicting tissue of origin for metastatic tumors with 88% accuracy. Additionally, we developed Deepcap, a cloud-based platform to make our deep learning model available to the scientific community. It predicts the cancer type of a given sample from the gene expression profile. It also performs unsupervised nonlinear dimensionality reduction to visualize input data in relation to TCGA data.

Our analysis resulted in the identification of a number of cancer biomarkers across different cancer types. These included several known markers confirming the validity of our approach. In addition, we identified several novel biomarkers that exhibited a cancer tissue-of-origin specific expression pattern. Such signatures are useful for the development of gene panels that can be used to screen and diagnose cancers. The model and gene signatures presented in this study can also be used to identify the tissue of origin for cancers of unknown primary (CUP). CUP accounts for 2–5% of all cancer diagnoses and represents a major area of unmet clinical need. Our list of cancer tissue-of-origin specific gene signatures can therefore be used for a number of applications including elucidation of novel therapeutic targets.

We compared cancer tissue-of-origin specific gene expression signatures identified in our study with the list of United States Food and Drug Administration (FDA)-approved gene expression-based in vitro cancer diagnostic tests (https://www.fda.gov/medical-devices/vitro-diagnostics/nucleic-acid-based-tests, accessed on 28 November 2020). There were only twelve genes that overlapped between our list and the FDA-approved marker list. All 12 genes were part of FDA-approved tests for breast cancer offered by Veridex, LLC (one gene), Agendia BV (three genes), and Nanostring Technologies (eight genes). However, only two of those 12 genes (i.e., SCGB2A2 and SCUBE2) were predicted as breast cancer signatures in our data set. The remaining 10 genes were part of the gene signature of other cancers. For example, MLPH and MIA genes were identified as signatures associated with UVM and SKCM. Both of these genes showed higher expression in UVM and SKCM compared to breast cancer, suggesting they are better markers for melanoma.

There are some limitations associated with our pan-cancer analysis-based assessment of cancer tissue-of-origin specific gene expression signatures [31]. For example, we only considered genes that were expressed at sufficiently high levels (≥5 FPKM) in at least 50% of samples within a cancer type. While this will limit the number of false-positive results, it may result in the loss of some markers that are expressed at low levels. We also focused only on genes that were overexpressed. Certain markers may be downregulated in specific cancer types, but they were not the focus of this study. Deep learning models aim to minimize the objective function. As a result, the model may not select certain genes that exhibit a cancer tissue-of-origin specific expression pattern if it has already identified many other candidate genes that yield better signals associated with that cancer type. This might help them achieve the study’s objective and minimize potential prediction errors. For example, although NAT1 is highly expressed in BRCA compared to other cancer types, our model did not select it. So, we expect some false negatives.

## 5. Conclusions

In conclusion, we implemented deep neural networks to accurately classify pan-cancer gene expression data. We then implemented the SHAP, a model agnostic method, to identify cancer tissue-of-origin specific gene signatures. The gene signatures showed a cancer tissue-of-origin specific expression pattern. In addition, the model based on gene signatures showed consistent performance during five-fold cross-validation. The cancer tissue-of-origin specific gene expression signatures identified through this pan-cancer analysis may represent valuable biomarkers and therapeutic targets that can guide future patient diagnosis and care. Moreover, the performance of the gene signature model was promising on ICGC metastatic data. In the future, it can be subjected to transfer learning using a large metastatic tumor gene expression data set to predict tissue of origin of CUPs.

## Data Availability

The DNN model is deployed on the cloud. Available online: www.deepcap.org. The source code is freely available at GitHub: www.github.com/MayurDivate/DeepCancerSignatures.

## Supplementary Materials

The following supporting information can be downloaded at: https://www.mdpi.com/article/10.3390/cancers14051185/s1, Figure S1: Model performed consistently during repeated training on randomly split data; Figure S2: Five-fold cross validation of model based on 976 gene signatures; Figure S3: Unsupervised clustering of TCGA RNA-seq data using log transformed FPKM values of 976 gene signatures; Figure S4: Heatmap showing expression of keratins used in diagnosis of BRCA, PRAD, CORE, and SKCM; Figure S5: Serverless architecture of the DCAP; Table S1: Total numbers of tumor samples and cancer codes of different cancer types used in this study; Table S2: Variance and maximum expression value of FPKM and log10 normalized data; Table S3: Table below shows model accuracy, precision, recall and f1-score during five-fold cross validation; and Table S4: List of gene signatures identified through interpretation of deep learning model.
