# Supplementary material for: Deep Learning-Based Pan-Cancer Classification Model Reveals Tissue-of-Origin Specific Gene Expression Signatures
Source: Cancers (Basel). 2022 Feb 24;14(5):1185. doi: 10.3390/cancers14051185 (PMC8909043; doi:10.3390/cancers14051185)
Supplement: Supplementary file 1 [file cancers-14-01185-s001.zip › cancers-1528320-supplementary .pdf]

Deep Learning-Based Pan-Cancer Classification Model Reveals Tissue-of-Origin Specific Gene Expression Signatures

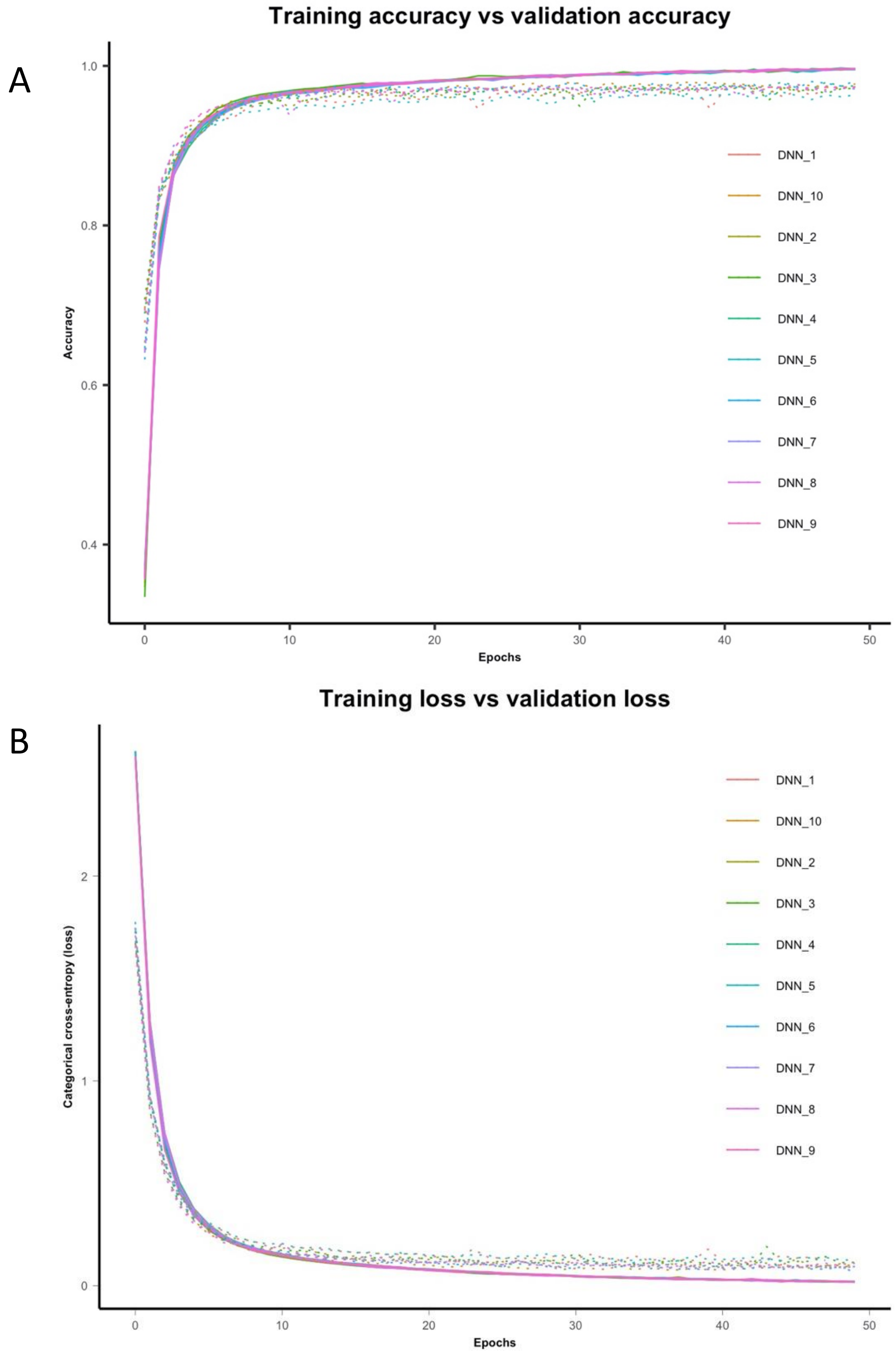

Figure S1. Model performed consistently during repeated training on randomly split data. Both A) accuracy and B) loss progress of model was consistent during repeated training of the model on randomly split 10 datasets.

A

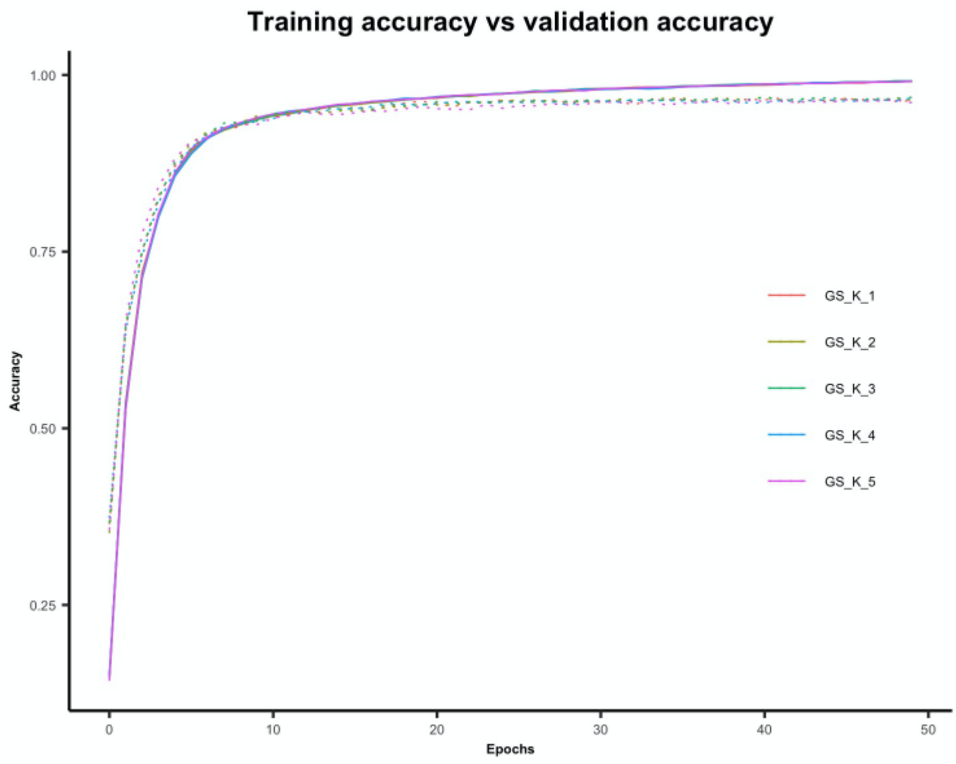

B

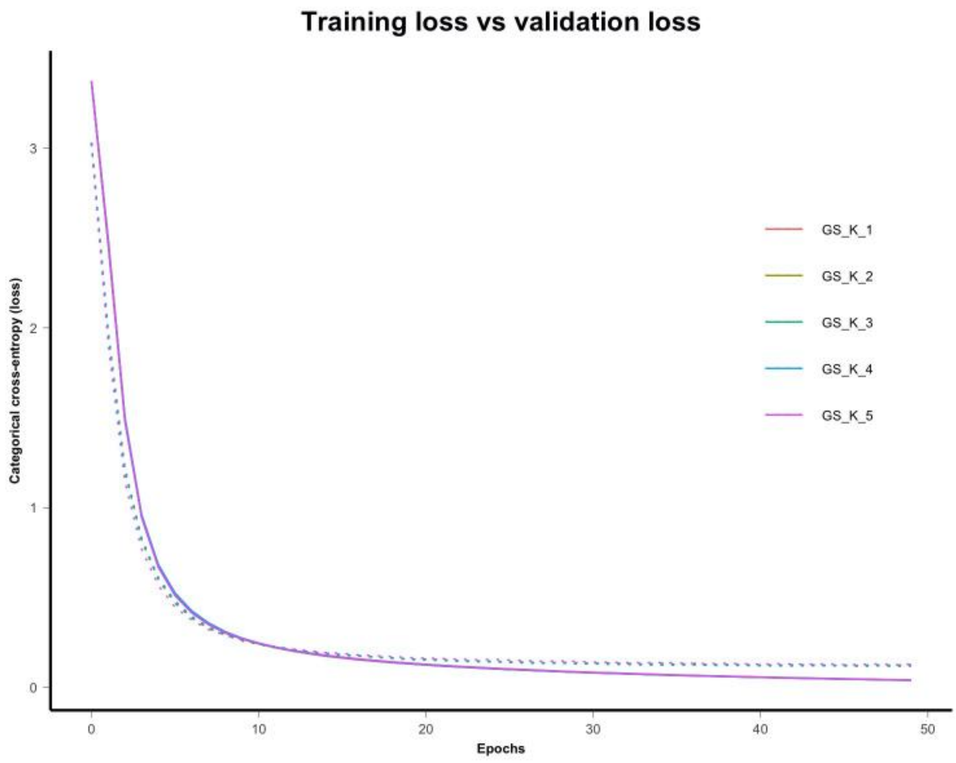

Figure S2. Five-fold cross validation of model based on 976 gene signatures. Performance of gene signatures based model remained stable and similar to original model during five-fold cross validation.

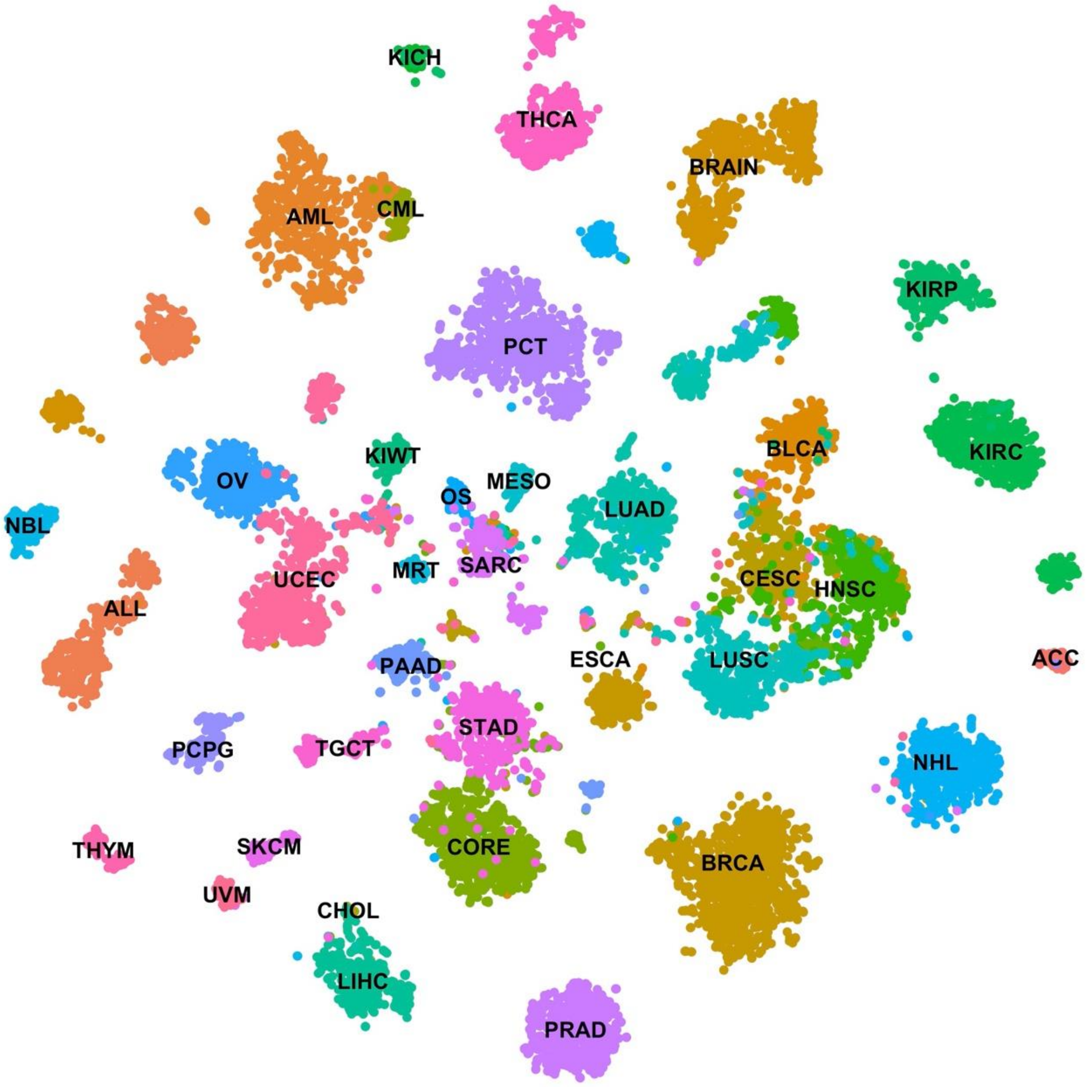

Figure S3. Unsupervised clustering of TCGA RNA-seq data using log transformed FPKM values of 976 gene signatures. Unsupervised clustering of TCGA data using 976 gene signatures provided distinct cancer clusters. Even subtypes such as UVM and SKCM formed well separated clusters.

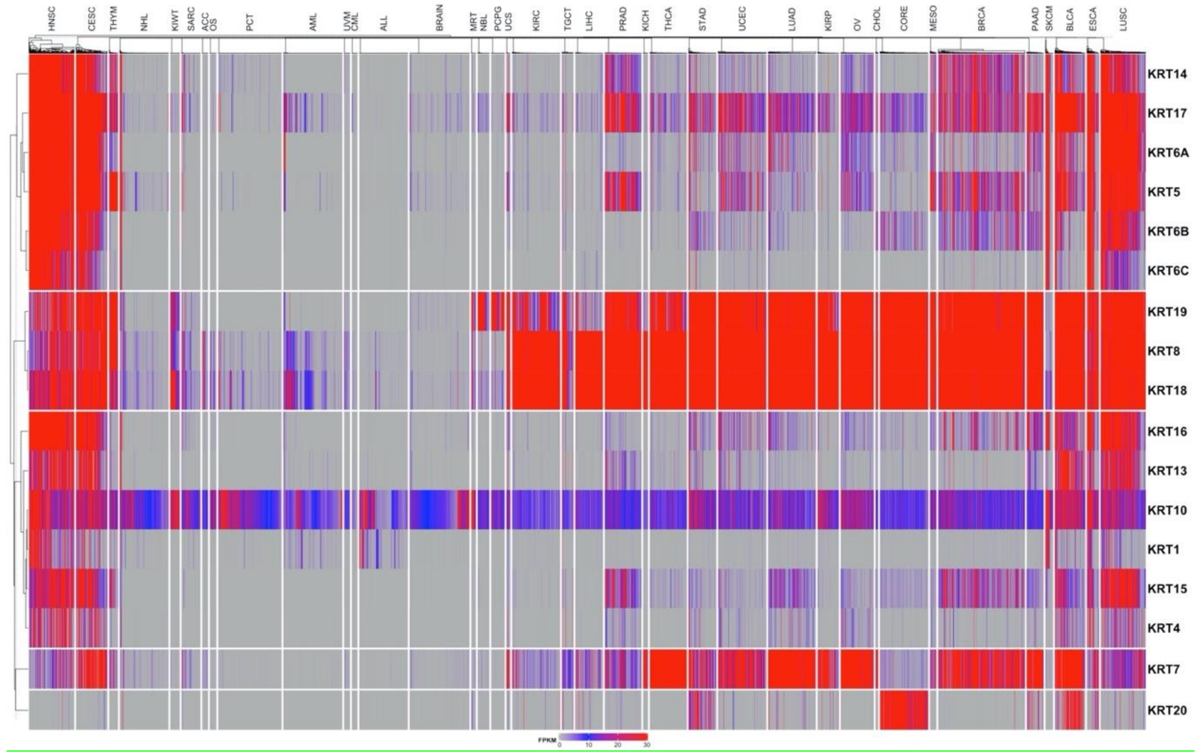

Figure S4. Heatmap showing expression of keratins used in diagnosis of BRCA, PRAD, CORE, and SKCM. As it can be seen from the heatmap that keratins used in the diagnosis cancer do not show cancer-specific expression.

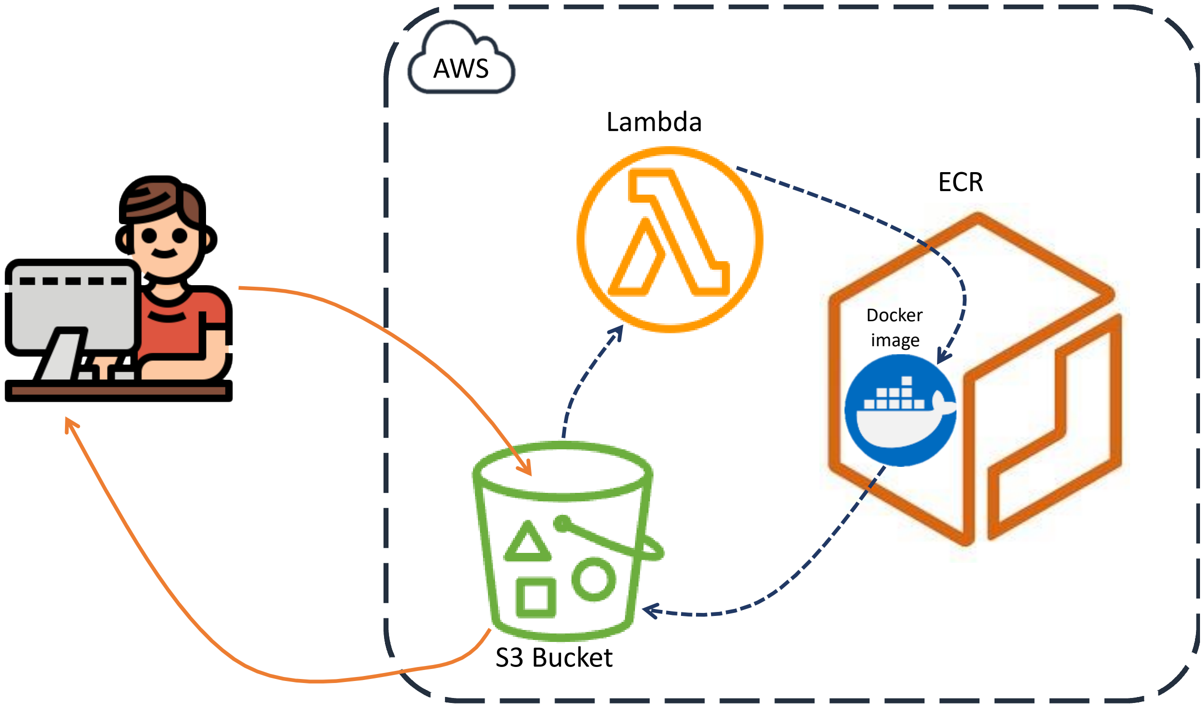

Figure S5. Serverless architecture of the DCAP. The user uses the web interface to upload the input CSV file. The data upload event initiates the lambda function, which then runs the ECR docker image to predict cancer type and perform t-SNE analysis. Finally, output files are saved to an S3 bucket, allowing the user to view and download the results

Table S1. Total numbers of tumor samples and cancer codes of different cancer types used in this study.

Sr. No.	Cancer type	Code	Samples
1.	Adrenocortical carcinoma	ACC	79
2.	Acute lymphoid leukemia	ALL	658
3.	Acute Myeloid Leukemia	AML	800
4.	Bladder Urothelial Carcinoma	BLCA	412
5.	Brain Cancer	BRAIN	812
6.	Breast invasive carcinoma	BRCA	1176
7.	Cervical squamous cell carcinoma and endocervical adenocarcinoma	CESC	426
8.	Cholangiocarcinoma	CHOL	34
9.	Chronic Myeloid Leukemia	CML	76
10.	Colorectal Cancer	CORE	655
11.	Esophageal carcinoma	ESCA	161
12.	Head and Neck squamous cell carcinoma	HNSC	610
13.	Kidney Chromophobe	KICH	65
14.	Kidney renal clear cell carcinoma	KIRC	649
15.	Kidney renal papillary cell carcinoma	KIRP	289
16.	Kidney Wilms tumor	KIWT	129
17.	Liver hepatocellular carcinoma	LIHC	374
18.	Lung adenocarcinoma	LUAD	646
19.	Lung squamous cell carcinoma	LUSC	611
20.	Mesothelioma	MESO	86

21.	Malignant rhabdoid tumor	MRT	62
22.	Neuroblastoma	NBL	160
23.	Non-Hodgkin's lymphoma	NHL	660
24.	Osteosarcoma	OS	88
25.	Ovarian serous cystadenocarcinoma	OV	450
26.	Pancreatic adenocarcinoma	PAAD	229
27.	Pheochromocytoma and Paragangliomas	PCPG	181
28.	Plasma Cell Tumors	PCT	859
29.	Prostate adenocarcinoma	PRAD	498
30.	Sarcoma	SARC	257
31.	Skin Cutaneous Melanoma	SKCM	103
32.	Stomach adenocarcinoma	STAD	376
33.	Testicular Germ Cell Tumors	TGCT	156
34.	Thyroid carcinoma	THCA	502
35.	Thymoma	THYM	119
36.	Uterine Corpus Endometrial Carcinoma	UCEC	709
37.	Uveal Melanoma	UVM	80
	Total number of samples		14237

Table S2. Variance and maximum expression value of FPKM and log10 normalized data.

Data	Variance	Max expression value
FPKM normalized data	23582909.87	141868.73
Log10 normalized data	1.41	5.15

Table S3. Table below shows model accuracy, precision, recall and f1-score during five-fold cross validation.

Fold number	Training accuracy (%)	Validation accuracy (%)	Test accuracy (%)	Precision (%)	Recall (%)	F1-score (%)
1	99.53	97.33	96.87	97.03	96.87	96.88
2	99.49	97.40	97.61	97.71	97.61	97.62
3	99.56	97.47	97.29	97.37	97.29	97.30
4	99.72	97.33	96.98	97.08	96.98	96.94
5	99.60	96.76	96.94	97.02	96.94	96.91

Table S4. List of gene signatures identified through interpretation of deep learning model.

Cancer	Gene Symbol	Occurrence frequency	Median FPKM	Enriched in cancers	Iteration
ACC	IGF2	8	1647.49	3	iter2
ACC	DLK1	10	1514.00	2	iter1
ACC	CYP17A1	9	1239.17	1	iter1
ACC	STAR	10	742.33	1	iter1
ACC	NPTX2	6	413.74	1	iter2
ACC	SERPINA5	6	324.33	2	iter1
ACC	CYP21A2	9	287.25	1	iter1
ACC	SULT2A1	9	272.68	2	iter1
ACC	CYP11A1	10	256.25	1	iter1
ACC	SNCG	7	241.88	2	iter2
ACC	MGARP	9	218.56	1	iter1
ACC	HSPB6	7	208.10	3	iter2
ACC	GSTA1	9	190.36	3	iter2
ACC	TUBB4A	7	154.12	5	iter2
ACC	NR5A1	10	135.24	1	iter1

ACC	MT3	6	109.59	2	iter2
ACC	TBX3	8	100.94	3	iter3
ACC	REEP6	5	89.75	5	iter2
ACC	CYP11B1	9	78.91	1	iter1
ACC	BEX1	6	64.74	5	iter2
ACC	FAM166B	5	61.07	1	iter2
ACC	INHA	5	56.10	1	iter2
ACC	KCNK3	8	55.63	1	iter1
ACC	PEG3	9	51.33	4	iter2
ACC	C7	5	49.59	5	iter1
ACC	CHGB	9	47.00	3	iter1
ACC	AGRP	9	43.61	1	iter2
ACC	GSTA3	9	34.89	1	iter1
ACC	CPB1	9	32.55	1	iter1
ACC	NR0B1	6	31.94	1	iter2
ACC	HSD3B2	9	25.92	1	iter1
ACC	PLA2G1B	5	23.07	1	iter1
ACC	AMHR2	9	22.82	1	iter1
ACC	FAM19A4	9	14.81	1	iter2
ACC	FAM43B	6	13.20	1	iter3
ALL	HBB	5	715.70	1	iter1
ALL	HIST1H1E	10	541.13	1	iter3
ALL	HIST1H4E	6	433.10	1	iter2
ALL	HIST1H1D	5	422.72	1	iter1
ALL	HIST1H3D	8	324.24	1	iter2
ALL	HIST1H3B	9	315.97	1	iter1
ALL	HIST2H2AC	10	275.52	1	iter3
ALL	HIST1H2BG	7	208.13	1	iter3
ALL	HIST1H1B	10	192.77	1	iter2
ALL	HIST1H2BH	5	188.82	1	iter1
ALL	HIST1H4C	7	184.71	1	iter1
ALL	HIST1H2BO	8	174.90	1	iter2
ALL	HIST1H2BF	10	172.60	1	iter3
ALL	HIST1H3F	10	124.29	1	iter2
ALL	HIST1H2AM	10	122.90	1	iter2
ALL	HIST1H3C	5	121.33	1	iter1
ALL	DNTT	10	120.16	2	iter1
ALL	HIST1H2AJ	10	117.24	1	iter2
ALL	HIST1H4D	6	117.06	1	iter1
ALL	HIST1H2AI	8	115.82	1	iter3
ALL	HIST1H2AH	10	111.21	1	iter3
ALL	HIST1H2BI	10	107.38	1	iter2
ALL	HIST2H2AB	9	103.75	1	iter3
ALL	HIST1H4B	5	102.94	1	iter1
ALL	HIST1H2BE	5	91.04	1	iter2
ALL	HIST1H3I	8	82.17	1	iter2
ALL	HIST1H4F	6	73.46	1	iter1
ALL	HIST1H2BL	10	69.89	1	iter3
ALL	HIST1H4A	9	69.11	1	iter1
ALL	HIST1H2AL	6	65.28	1	iter2
ALL	AL138751.1	8	64.70	1	iter3
ALL	C1orf228	9	54.02	3	iter3
ALL	HIST1H2AB	10	52.62	1	iter3
ALL	IGLL1	9	51.36	3	iter1
ALL	HIST1H2BB	7	46.49	1	iter1
ALL	HIST1H4L	8	36.73	1	iter2

ALL	TNF	9	27.03	1	iter1
ALL	HBA1	10	26.82	1	iter1
ALL	VPREB1	10	22.04	1	iter1
ALL	HIST1H3J	10	18.66	1	iter3
ALL	HIST1H3G	5	15.41	1	iter3
ALL	AHSP	5	14.02	1	iter3
ALL	ARPP21	7	11.05	2	iter2
AML	MPO	10	502.61	2	iter1
AML	RNASE2	10	260.27	2	iter1
AML	AZU1	10	192.89	2	iter1
AML	PRSS57	10	132.96	1	iter1
AML	ELANE	10	129.44	2	iter1
AML	PRTN3	10	95.23	2	iter1
AML	CTSG	5	71.01	2	iter2
AML	S100A12	7	62.29	3	iter1
AML	FLT3	10	53.32	2	iter1
AML	RNASE3	10	52.47	2	iter1
AML	NFE2	5	44.53	2	iter3
AML	PRAM1	9	41.55	2	iter2
AML	PLAC8	9	37.32	4	iter3
AML	P2RX1	9	35.95	4	iter3
AML	RAB37	7	26.47	3	iter3
AML	MTRNR2L12	10	25.00	3	iter1
AML	SPINK2	7	23.23	1	iter1
AML	C11orf21	5	22.01	2	iter3
AML	MLC1	9	20.45	2	iter1
AML	MS4A3	10	19.76	2	iter2
AML	GATA2	6	19.48	5	iter3
AML	TSPAN32	5	16.78	2	iter3
AML	RAB44	10	16.24	2	iter2
AML	GAPT	6	15.93	2	iter2
AML	SPNS3	5	15.69	1	iter3
AML	CLC	7	14.82	2	iter3
AML	HOXA9	5	14.25	3	iter1
AML	VSTM1	6	13.57	2	iter2
AML	PADI4	6	12.63	2	iter2
AML	TPSAB1	7	10.74	1	iter3
AML	HBD	6	10.55	2	iter1
AML	MTRNR2L8	10	10.50	2	iter1
BLCA	KRT17	8	503.82	5	iter1
BLCA	S100P	6	390.51	5	iter2
BLCA	SNCG	7	190.09	2	iter2
BLCA	UPK2	7	98.03	1	iter1
BLCA	GATA3	9	64.54	4	iter1
BLCA	UPK1B	7	62.30	2	iter1
BLCA	VGLL1	7	37.84	1	iter1
BLCA	DHRS2	7	37.83	1	iter1
BLCA	UPK1A	8	31.15	1	iter1
BLCA	KRT20	7	12.31	2	iter1
BLCA	UPK3A	9	10.68	2	iter2
BLCA	ACTG2	7	10.53	5	iter1
BRAIN	GFAP	10	2270.33	1	iter1
BRAIN	BCAN	9	529.82	1	iter1
BRAIN	PMP2	10	233.64	1	iter1
BRAIN	GPM6B	10	227.89	3	iter3
BRAIN	AGT	5	219.83	3	iter2

BRAIN	PTPRZ1	9	170.00	1	iter3
BRAIN	AQP4	8	167.84	1	iter1
BRAIN	OLIG1	7	164.16	1	iter2
BRAIN	GPM6A	8	133.25	1	iter1
BRAIN	BAALC	6	116.81	1	iter1
BRAIN	MLC1	5	111.02	2	iter1
BRAIN	SOX8	7	92.31	1	iter2
BRAIN	C1QL1	8	89.39	4	iter3
BRAIN	C1orf61	6	81.99	1	iter1
BRAIN	ADCYAP1R1	7	80.01	1	iter1
BRAIN	OLIG2	10	73.35	1	iter1
BRAIN	NCAN	10	69.72	1	iter2
BRAIN	NCAM1	10	61.40	4	iter2
BRAIN	TTYH1	5	58.45	1	iter2
BRAIN	CTNND2	10	58.17	1	iter1
BRAIN	SLC1A2	9	52.48	1	iter3
BRAIN	PHYHIPL	6	46.52	3	iter2
BRAIN	TIMP4	5	40.31	1	iter2
BRAIN	CACNG7	5	36.15	2	iter1
BRAIN	GPR37L1	6	33.28	1	iter2
BRAIN	TNR	9	30.90	1	iter1
BRAIN	LHFPL3	8	30.29	1	iter2
BRAIN	HEPACAM	10	28.99	1	iter2
BRAIN	SCRG1	8	25.52	1	iter3
BRAIN	GRIA3	6	17.69	1	iter2
BRAIN	NDP	5	17.12	1	iter3
BRAIN	C8orf46	10	16.89	1	iter3
BRAIN	MAG	9	15.96	1	iter1
BRAIN	AMER2	9	14.81	1	iter2
BRAIN	BRINP2	10	14.52	1	iter3
BRAIN	RFX4	8	14.02	1	iter1
BRAIN	GABBR2	8	11.59	1	iter3
BRAIN	MOG	5	10.75	1	iter1
BRCA	MGP	9	450.03	3	iter2
BRCA	AZGP1	10	263.54	3	iter1
BRCA	SFRP2	10	195.86	4	iter2
BRCA	POSTN	5	183.96	5	iter2
BRCA	GATA3	10	163.90	4	iter1
BRCA	SPDEF	7	92.15	3	iter1
BRCA	PIP	10	42.56	1	iter1
BRCA	TRPS1	10	40.59	1	iter1
BRCA	KIAA1324	7	40.33	3	iter2
BRCA	SCGB2A2	10	32.59	1	iter1
BRCA	LTF	7	26.76	4	iter1
BRCA	STC2	9	26.37	2	iter2
BRCA	COMP	5	25.81	4	iter3
BRCA	ZG16B	8	25.73	3	iter3
BRCA	SCUBE2	5	19.22	2	iter2
BRCA	IRX5	9	18.11	1	iter1
BRCA	PRLR	10	16.97	1	iter1
BRCA	LRRC15	6	16.60	2	iter3
BRCA	CILP	10	16.59	1	iter2
BRCA	AR	9	13.92	2	iter3
BRCA	LMX1B	10	10.88	1	iter1
CESC	KRT5	7	1697.32	5	iter1
CESC	KRT6A	9	1097.52	4	iter1

CESC	KRT17	7	1093.99	5	iter1
CESC	S100A8	5	718.38	4	iter3
CESC	KRT13	9	110.45	4	iter2
CESC	SERPINB3	10	90.73	2	iter1
CESC	CALML3	9	88.07	5	iter1
CESC	CXCL17	6	83.83	4	iter1
CESC	S100A7	7	54.46	4	iter1
CESC	SERPINB4	10	49.76	1	iter1
CESC	CDKN2A	10	47.86	4	iter1
CESC	LGALS7B	10	40.20	2	iter1
CESC	EPHX3	7	23.49	1	iter1
CESC	SERPINB13	8	22.17	3	iter2
CHOL	CRP	7	800.61	2	iter1
CHOL	SERPINA1	6	732.85	5	iter3
CHOL	DEFB1	6	701.71	3	iter3
CHOL	TM4SF4	6	565.04	2	iter1
CHOL	VTN	9	435.23	4	iter1
CHOL	APCS	8	274.01	2	iter1
CHOL	FGG	6	116.45	2	iter1
CHOL	GJB1	5	81.87	5	iter2
CHOL	DCDC2	5	80.68	1	iter1
CHOL	HABP2	6	70.94	2	iter2
CHOL	ASGR2	5	46.25	2	iter3
CHOL	ANXA13	7	44.87	1	iter2
CHOL	SFRP5	6	36.80	1	iter1
CHOL	ITIH5	5	35.86	1	iter2
CHOL	SCTR	5	32.54	1	iter2
CHOL	CLDN10	6	29.91	2	iter1
CHOL	CXCL6	5	25.78	1	iter1
CHOL	EPS8L3	6	21.60	4	iter2
CHOL	CHST4	6	12.97	1	iter3
CHOL	CFTR	5	12.93	4	iter3
CHOL	POF1B	5	11.20	5	iter2
CML	S100A9	10	21819.00	1	iter3
CML	S100A8	6	3759.62	4	iter3
CML	CXCL8	6	873.72	1	iter1
CML	G0S2	6	785.62	1	iter2
CML	SPI1	6	515.25	1	iter1
CML	ELANE	6	510.13	2	iter1
CML	PGLYRP1	6	504.41	1	iter1
CML	DEFA3	6	437.83	1	iter2
CML	MMP8	7	369.72	1	iter2
CML	DEFA4	9	340.83	1	iter3
CML	PRTN3	5	266.68	2	iter1
CML	FCN1	5	261.69	1	iter1
CML	CEACAM8	9	237.22	1	iter1
CML	BPI	7	232.77	1	iter1
CML	FCGR3B	8	190.50	1	iter2
CML	CTSG	7	179.73	2	iter2
CML	GPR97	6	161.11	1	iter1
CML	MMP25	6	149.24	1	iter1
CML	CD177	7	120.35	1	iter1
CML	CRISP3	5	112.04	1	iter3
CML	ARG1	5	108.80	2	iter3
CML	MGAM	10	59.63	1	iter3
CML	OLFM4	6	53.35	4	iter2

CML	CYP4F3	6	48.66	2	iter1
CML	CXCR2	7	48.61	1	iter3
CML	IL18RAP	5	47.42	1	iter3
CML	MTRNR2L12	7	45.54	3	iter1
CML	RGL4	7	45.23	1	iter1
CML	GLT1D1	9	42.66	1	iter2
CML	CEACAM3	9	32.65	1	iter1
CML	CMTM2	5	23.77	1	iter3
CML	SERPINB10	6	23.18	1	iter2
CML	PF4	6	19.16	1	iter2
CML	MTRNR2L8	8	18.73	2	iter1
CML	EMR3	6	18.61	1	iter2
CORE	CEACAM5	10	913.58	1	iter1
CORE	TFF3	6	472.35	1	iter1
CORE	GPX2	7	446.06	5	iter2
CORE	LGALS4	9	397.63	4	iter1
CORE	TSPAN8	8	241.29	3	iter1
CORE	MUC13	7	176.18	2	iter1
CORE	CDH17	6	148.33	1	iter1
CORE	CDX1	10	120.56	1	iter1
CORE	PHGR1	7	118.19	2	iter2
CORE	FAM3D	6	107.52	1	iter2
CORE	FABP1	9	89.93	2	iter1
CORE	C10orf99	5	73.31	2	iter2
CORE	GPA33	9	70.75	1	iter1
CORE	DPEP1	8	69.20	1	iter1
CORE	NOX1	10	65.77	1	iter1
CORE	PRSS3	7	63.27	4	iter1
CORE	CDX2	8	61.69	1	iter1
CORE	ASCL2	5	57.80	1	iter2
CORE	VIL1	5	54.90	3	iter1
CORE	EPS8L3	8	49.97	4	iter2
CORE	HOXB9	9	49.28	3	iter3
CORE	HNF4A	8	43.29	4	iter2
CORE	CLRN3	10	42.21	5	iter3
CORE	POF1B	7	41.46	5	iter2
CORE	CCL24	6	40.77	1	iter1
CORE	FUT3	7	40.31	3	iter3
CORE	RNF43	8	39.81	1	iter2
CORE	SPINK4	10	32.88	1	iter1
CORE	AXIN2	8	28.90	1	iter3
CORE	CEACAM7	6	27.78	1	iter2
CORE	MEP1A	8	27.35	1	iter2
CORE	GUCY2C	7	22.39	1	iter3
CORE	TRIM31	5	21.14	2	iter2
CORE	MMP12	7	20.53	5	iter3
CORE	LEFTY1	7	18.79	1	iter3
CORE	SLC26A3	6	16.02	1	iter3
CORE	HOXB8	8	13.80	3	iter2
CORE	TRABD2A	5	13.63	1	iter3
CORE	TRIM15	5	13.08	1	iter3
ESCA	SPRR1B	10	192.64	4	iter2
ESCA	KRT14	7	152.87	5	iter1
ESCA	SPRR1A	5	54.55	5	iter1
ESCA	DSC3	7	28.51	4	iter2
ESCA	SPRR2A	5	28.20	4	iter2

ESCA	CALML3	8	24.30	5	iter1
ESCA	RHCG	5	16.62	4	iter1
ESCA	SPRR3	5	14.80	5	iter1
ESCA	HR	5	13.19	2	iter2
ESCA	CCL20	6	12.75	5	iter2
HNSC	KRT14	10	4117.88	5	iter1
HNSC	KRT5	10	2870.63	5	iter1
HNSC	KRT17	8	2330.19	5	iter1
HNSC	SPRR1A	6	672.59	5	iter1
HNSC	SPRR2A	5	288.83	4	iter2
HNSC	PKP1	6	245.39	4	iter3
HNSC	SPRR2E	7	201.66	1	iter3
HNSC	KRT6C	8	183.81	1	iter2
HNSC	COL17A1	5	166.04	2	iter1
HNSC	MMP1	6	159.56	3	iter1
HNSC	KRTDAP	5	153.76	1	iter3
HNSC	FGFBP1	7	153.48	2	iter2
HNSC	CALML3	9	121.08	5	iter1
HNSC	DSC3	10	99.94	4	iter2
HNSC	KRT13	9	98.82	4	iter2
HNSC	IVL	6	80.57	2	iter3
HNSC	LGALS7B	10	80.27	2	iter1
HNSC	CALML5	10	53.76	2	iter1
HNSC	PTHLH	8	41.74	2	iter3
HNSC	TGM1	6	38.74	3	iter3
HNSC	KRT15	7	35.44	5	iter1
HNSC	GBP6	6	28.21	2	iter3
HNSC	MMP10	7	27.62	2	iter1
HNSC	MMP3	10	26.08	2	iter1
HNSC	BNC1	8	24.84	3	iter2
HNSC	FAT2	6	21.70	3	iter3
HNSC	TMPRSS11D	6	18.88	1	iter1
HNSC	IL36G	7	18.68	1	iter2
HNSC	DSG1	8	17.33	1	iter2
HNSC	RRAD	6	14.08	3	iter2
HNSC	HSPA2	8	13.16	5	iter2
HNSC	IL36RN	5	11.97	1	iter3
HNSC	HEPHL1	8	11.40	1	iter2
KICH	PVALB	9	573.86	1	iter1
KICH	HSD11B2	6	502.87	1	iter2
KICH	ATP6V0D2	9	399.15	1	iter1
KICH	CA12	5	396.89	1	iter3
KICH	MAL	6	316.57	4	iter3
KICH	FOXI1	9	256.14	1	iter1
KICH	ATP6V1B1	10	251.43	2	iter1
KICH	CLCNKB	8	248.86	1	iter1
KICH	KLK1	9	216.88	1	iter1
KICH	ATP6V0A4	9	209.58	1	iter1
KICH	TMEM213	9	191.38	1	iter1
KICH	SLC4A1	7	152.94	1	iter2
KICH	ERP27	7	136.94	1	iter3
KICH	CLDN8	10	104.55	1	iter1
KICH	HEPACAM2	6	102.50	1	iter2
KICH	DMRT2	8	101.51	1	iter2
KICH	ATP6V1G3	6	101.47	1	iter1
KICH	STAP1	8	99.69	3	iter2

KICH	TRIM50	8	60.90	1	iter1
KICH	PTGER3	9	49.85	1	iter1
KICH	RHBG	7	46.62	1	iter2
KICH	TMEM72	8	39.10	1	iter3
KICH	BSND	9	35.13	1	iter1
KICH	FGF9	7	19.90	1	iter3
KICH	TMEM52B	5	19.61	1	iter2
KICH	GCGR	5	19.42	1	iter1
KICH	HOXB9	8	14.75	3	iter3
KICH	HOXD8	5	12.22	4	iter2
KICH	LRRC14B	6	10.86	1	iter3
KICH	SFTPB	6	10.31	4	iter1
KIRC	NDUFA4L2	7	467.64	1	iter1
KIRC	NAT8	10	202.85	3	iter1
KIRC	GSTA1	6	148.71	3	iter2
KIRC	RBP5	5	125.52	3	iter2
KIRC	CA9	9	113.63	2	iter2
KIRC	UGT2B7	10	88.13	3	iter1
KIRC	ENPP3	7	80.13	1	iter3
KIRC	BHMT2	5	60.68	3	iter1
KIRC	SLC3A1	8	60.52	2	iter2
KIRC	BHMT	9	60.11	2	iter1
KIRC	CLDN2	10	57.92	4	iter2
KIRC	ENPEP	7	55.99	1	iter1
KIRC	GSTA2	6	47.26	2	iter1
KIRC	SLC28A1	9	43.80	2	iter1
KIRC	PDZK1	7	42.67	4	iter2
KIRC	UGT2A3	5	42.53	3	iter2
KIRC	FXYD2	9	34.97	3	iter1
KIRC	LRP2	9	32.25	2	iter1
KIRC	CDHR5	5	31.91	5	iter3
KIRC	SLC6A13	6	31.18	2	iter1
KIRC	SLC22A2	9	30.89	2	iter2
KIRC	CUBN	7	30.52	1	iter2
KIRC	SLC6A3	9	28.41	1	iter1
KIRC	SEMA5B	8	24.93	1	iter2
KIRC	C14orf105	7	19.22	2	iter1
KIRC	SLC16A12	6	18.56	2	iter3
KIRC	UGT1A9	5	16.17	1	iter1
KIRC	DMGDH	5	15.75	2	iter3
KIRC	ACSM2B	8	15.55	2	iter3
KIRC	DPYS	5	14.48	2	iter3
KIRC	FABP7	8	13.76	2	iter1
KIRC	NR1H4	5	13.31	3	iter3
KIRC	AGXT2	8	12.64	1	iter3
KIRC	RNF186	5	11.52	2	iter3
KIRC	SLC22A11	6	11.22	2	iter2
KIRC	PNCK	8	10.21	1	iter3
KIRP	CRYAB	7	1013.50	2	iter2
KIRP	PIGR	7	419.25	3	iter2
KIRP	TMEM27	5	121.40	2	iter1
KIRP	NAT8	10	112.84	3	iter1
KIRP	TFPI2	5	88.95	1	iter3
KIRP	UGT2B7	9	87.19	3	iter1
KIRP	CLDN2	7	77.66	4	iter2
KIRP	CYS1	5	74.27	2	iter2

KIRP	CDH16	8	63.60	3	iter1
KIRP	KCNJ16	9	57.88	3	iter1
KIRP	PDZK1	7	56.17	4	iter2
KIRP	SLC3A1	8	54.67	2	iter2
KIRP	HNF1B	7	54.28	3	iter1
KIRP	LYG1	5	42.81	2	iter2
KIRP	MIOX	8	41.00	2	iter2
KIRP	GLYAT	6	36.92	3	iter1
KIRP	TMEM252	6	36.92	1	iter3
KIRP	BBOX1	8	35.59	3	iter1
KIRP	C14orf105	8	35.38	2	iter1
KIRP	ACSM2A	5	29.76	3	iter1
KIRP	AOC1	7	28.18	5	iter3
KIRP	POU3F3	5	26.16	4	iter1
KIRP	SLC17A1	8	24.26	2	iter1
KIRP	CLRN3	5	24.16	5	iter3
KIRP	LRRN4	5	23.69	3	iter1
KIRP	KCNJ15	7	22.08	3	iter3
KIRP	ACMSD	5	21.09	3	iter1
KIRP	SMTNL2	5	17.91	2	iter1
KIRP	HOGA1	7	15.98	1	iter2
KIRP	KL	6	15.27	2	iter2
KIRP	PPP1R1A	8	14.34	1	iter3
KIRP	TRABD2B	7	14.33	2	iter2
KIRP	HOXA5	5	13.36	4	iter3
KIRP	SPX	8	12.95	2	iter3
KIRP	SLC17A3	6	11.32	2	iter1
KIWT	IGF2	5	2543.73	3	iter2
KIWT	NNAT	10	299.82	2	iter1
KIWT	SIX2	5	241.57	1	iter1
KIWT	PEG10	8	159.68	2	iter2
KIWT	UNCX	10	138.51	1	iter1
KIWT	PAX2	10	134.53	1	iter1
KIWT	COL2A1	7	111.68	1	iter1
KIWT	SALL1	10	104.59	1	iter1
KIWT	SHISA3	6	86.87	1	iter3
KIWT	HOXC10	6	85.91	2	iter2
KIWT	LYPD1	6	84.23	2	iter1
KIWT	CNTFR	9	82.96	3	iter1
KIWT	SHISA2	6	80.16	1	iter1
KIWT	EYA1	8	78.70	1	iter1
KIWT	DACH1	7	67.62	1	iter1
KIWT	MEOX1	7	57.34	1	iter1
KIWT	FOXC2	10	54.49	1	iter2
KIWT	WASF3	8	50.87	2	iter1
KIWT	HAS2	6	48.20	1	iter3
KIWT	FOXC1	5	43.39	4	iter3
KIWT	HOXA11	6	43.31	1	iter3
KIWT	MYCN	5	38.41	3	iter2
KIWT	CITED1	10	34.79	4	iter1
KIWT	DAPL1	5	34.31	4	iter2
KIWT	HOXD11	5	29.94	1	iter1
KIWT	C1QTNF4	6	28.13	3	iter1
KIWT	GFRA1	8	27.42	2	iter1
KIWT	TRABD2B	10	25.65	2	iter2
KIWT	FOXD1	5	17.82	1	iter2

KIWT	GPR64	6	17.62	1	iter2
KIWT	FGF8	6	15.64	1	iter1
KIWT	CDO1	6	15.17	3	iter2
KIWT	BMPER	7	15.00	1	iter2
KIWT	TMEM100	5	13.93	2	iter3
KIWT	GDNF	5	13.88	1	iter3
KIWT	BRINP1	5	11.24	1	iter2
KIWT	PEG3	5	10.46	4	iter2
KIWT	TCF21	5	10.23	2	iter3
KIWT	SHISA8	8	10.12	1	iter2
LIHC	ALB	10	12188.40	1	iter1
LIHC	APOA2	8	9313.44	1	iter1
LIHC	APOC3	5	3144.20	1	iter1
LIHC	RBP4	9	2946.14	1	iter3
LIHC	APOH	8	2785.61	1	iter1
LIHC	AMBP	10	2440.03	1	iter1
LIHC	VTN	6	2033.85	4	iter1
LIHC	FGA	6	2017.55	1	iter1
LIHC	FGB	5	1778.57	1	iter1
LIHC	ORM1	6	1721.49	1	iter1
LIHC	SERPINC1	10	1282.02	1	iter2
LIHC	FGG	5	1193.44	2	iter1
LIHC	AHSG	6	977.85	1	iter3
LIHC	GC	10	904.57	1	iter1
LIHC	HP	9	855.16	1	iter2
LIHC	TF	9	641.75	1	iter3
LIHC	ORM2	8	633.50	1	iter1
LIHC	APCS	7	622.77	2	iter1
LIHC	KNG1	9	544.85	1	iter2
LIHC	ITIH2	10	538.00	1	iter1
LIHC	F2	7	486.78	1	iter2
LIHC	HPX	8	459.99	1	iter3
LIHC	ITIH1	8	313.57	1	iter1
LIHC	SERPIND1	8	309.86	1	iter2
LIHC	HRG	5	303.85	1	iter3
LIHC	APOB	10	281.11	1	iter2
LIHC	CPB2	10	242.98	1	iter1
LIHC	UGT2B4	8	240.31	1	iter2
LIHC	FGL1	10	231.24	1	iter2
LIHC	ITIH3	6	230.91	1	iter2
LIHC	LBP	7	229.77	1	iter1
LIHC	IGFBP1	8	228.68	1	iter1
LIHC	C8G	6	213.56	1	iter2
LIHC	SERPINA6	7	208.72	2	iter2
LIHC	AGXT	7	198.34	1	iter3
LIHC	MAT1A	5	188.64	1	iter3
LIHC	ASGR2	7	182.54	2	iter3
LIHC	CRP	10	154.48	2	iter1
LIHC	PLG	7	145.77	1	iter2
LIHC	CFHR1	9	145.43	1	iter2
LIHC	BAAT	10	136.69	1	iter2
LIHC	ADH1A	5	131.61	1	iter3
LIHC	SLC38A3	5	121.81	1	iter3
LIHC	TFR2	6	121.34	1	iter3
LIHC	PROC	8	112.70	1	iter3
LIHC	C8B	9	93.80	1	iter2

LIHC	G6PC	6	89.10	1	iter3
LIHC	HAO1	6	82.75	1	iter3
LIHC	AKR1C4	6	82.64	1	iter3
LIHC	CYP2E1	5	74.76	1	iter2
LIHC	HFE2	5	70.97	1	iter3
LIHC	F13B	9	39.38	1	iter3
LIHC	F7	8	37.66	1	iter3
LIHC	PGLYRP2	6	25.39	1	iter3
LIHC	FGF21	5	10.35	1	iter3
LUAD	SFTPB	10	989.92	4	iter1
LUAD	SFTPA2	10	503.12	2	iter1
LUAD	CEACAM6	5	425.91	4	iter1
LUAD	SFTPA1	10	395.72	2	iter1
LUAD	SLC34A2	10	322.30	4	iter1
LUAD	NAPSA	10	296.90	3	iter1
LUAD	SFTA2	6	143.77	1	iter1
LUAD	CXCL17	6	131.56	4	iter1
LUAD	SFTPC	10	62.80	1	iter1
LUAD	SFTPD	9	62.70	2	iter1
LUAD	SCGB3A2	10	55.32	3	iter1
LUAD	NKX2-1	9	50.07	2	iter1
LUAD	C16orf89	10	46.03	3	iter2
LUAD	CCL18	6	43.67	5	iter2
LUAD	C4BPA	5	41.54	3	iter1
LUAD	SCGB3A1	10	40.92	1	iter1
LUAD	CP	5	37.35	5	iter2
LUAD	CTSE	5	34.92	3	iter1
LUAD	AGR3	8	34.59	5	iter2
LUAD	SFTA3	10	27.95	2	iter1
LUAD	CLIC6	7	25.41	3	iter3
LUAD	SLC22A31	10	25.26	2	iter2
LUAD	SCGB1A1	9	20.54	2	iter1
LUAD	MARCO	8	16.91	1	iter3
LUAD	NDNF	10	16.10	3	iter2
LUAD	CAPN8	7	16.04	2	iter2
LUAD	ST6GALNAC1	8	15.88	5	iter2
LUAD	SDR16C5	5	13.68	2	iter2
LUAD	FAM83A	5	11.50	3	iter2
LUAD	CYP4B1	6	10.89	2	iter3
LUAD	BPIFB1	9	10.65	1	iter2
LUAD	SCNN1B	5	10.12	1	iter2
LUAD	CCL13	5	10.09	1	iter1
LUSC	KRT6A	10	975.03	4	iter1
LUSC	KRT5	9	962.51	5	iter1
LUSC	SFTPB	10	125.14	4	iter1
LUSC	KRT16	7	104.72	4	iter2
LUSC	SFTPA2	10	95.92	2	iter1
LUSC	CALML3	8	69.84	5	iter1
LUSC	SFTPA1	10	66.03	2	iter1
LUSC	KRT6B	8	64.25	5	iter2
LUSC	TP63	5	61.53	5	iter3
LUSC	DSG3	5	59.46	4	iter1
LUSC	MMP12	8	35.51	5	iter3
LUSC	PTHLH	7	25.32	2	iter3
LUSC	NTS	10	20.57	1	iter2
LUSC	SCGB3A2	10	17.04	3	iter1

LUSC	SPRR2D	5	16.55	4	iter2
LUSC	DAPL1	8	16.48	4	iter2
LUSC	CES1	5	15.93	3	iter3
LUSC	SFTPD	10	13.69	2	iter1
LUSC	NAPSA	10	12.39	3	iter1
LUSC	SCGB1A1	10	10.76	2	iter1
LUSC	EGFL6	10	10.49	1	iter2
MESO	ITLN1	10	854.58	1	iter1
MESO	UPK3B	10	357.66	3	iter1
MESO	CALB2	6	344.11	1	iter1
MESO	MSLN	7	257.88	3	iter2
MESO	UPK1B	9	98.87	2	iter1
MESO	SAA1	6	87.85	4	iter2
MESO	PRG4	8	66.49	1	iter2
MESO	VTN	10	55.97	4	iter1
MESO	CPA4	9	55.30	1	iter1
MESO	HAS1	10	45.67	1	iter1
MESO	MYRF	6	39.39	4	iter1
MESO	BNC1	7	34.17	3	iter2
MESO	ANXA8	6	25.91	3	iter2
MESO	PDIA2	5	22.29	2	iter1
MESO	RSPO1	7	21.64	1	iter2
MESO	FAM101A	6	21.64	2	iter2
MESO	TMEM151A	8	21.37	2	iter1
MESO	INMT	5	18.31	1	iter2
MESO	ITLN2	10	18.21	1	iter1
MESO	GALNT9	8	10.36	1	iter2
MRT	MEST	5	390.38	2	iter1
MRT	TNNT1	6	117.08	5	iter2
MRT	CDC42EP5	8	93.97	4	iter3
MRT	APLP1	10	62.86	4	iter1
MRT	PCDH18	5	31.94	3	iter1
MRT	NEFH	7	17.60	4	iter2
MRT	COL26A1	6	14.02	2	iter1
MRT	NPW	7	11.95	2	iter1
MRT	CACNG7	8	11.33	2	iter1
MRT	SLC17A7	9	10.46	1	iter1
NBL	STMN2	10	807.06	2	iter1
NBL	DBH	7	390.10	2	iter1
NBL	TUBB2B	5	299.20	2	iter2
NBL	CHGB	7	232.16	3	iter1
NBL	GAP43	10	212.39	2	iter1
NBL	HAND2	6	196.31	2	iter1
NBL	TH	8	173.04	2	iter1
NBL	PHOX2B	10	149.57	2	iter1
NBL	PHOX2A	7	145.65	2	iter1
NBL	HMP19	5	97.97	2	iter1
NBL	GNG3	10	95.80	1	iter1
NBL	RTN1	9	85.28	2	iter3
NBL	STMN4	10	84.86	1	iter1
NBL	PRPH	10	77.01	1	iter1
NBL	RUNDC3A	9	72.47	2	iter3
NBL	ISL1	10	72.42	1	iter2
NBL	CELF3	6	62.51	2	iter2
NBL	MAB21L2	10	55.87	2	iter1
NBL	ELAVL4	10	47.86	1	iter2

NBL	GFRA3	8	46.36	1	iter3
NBL	ELAVL3	8	42.41	2	iter2
NBL	MAB21L1	5	40.35	2	iter2
NBL	GDAP1L1	9	39.68	1	iter2
NBL	ACTL6B	8	38.50	2	iter3
NBL	ATCAY	5	38.27	3	iter3
NBL	C1QL1	6	37.29	4	iter3
NBL	HAND1	8	37.08	2	iter1
NBL	CADM3	5	37.00	2	iter3
NBL	KCNQ2	9	33.94	3	iter2
NBL	NTRK1	9	32.63	2	iter3
NBL	Mar-11	7	31.24	1	iter1
NBL	TTC9B	9	30.36	1	iter1
NBL	INSM2	9	29.26	1	iter1
NBL	NEFM	9	29.00	2	iter1
NBL	NEFL	5	27.81	3	iter1
NBL	HMX1	9	25.96	1	iter2
NBL	SNAP91	7	25.20	2	iter2
NBL	NRSN1	7	25.04	1	iter1
NBL	VSTM2A	7	23.21	2	iter3
NBL	NXPH1	7	21.98	1	iter1
NBL	DCX	7	21.37	1	iter2
NBL	TLX2	8	19.09	2	iter2
NBL	FEV	6	17.98	2	iter3
NBL	FAM131B	5	15.11	2	iter3
NBL	P2RX3	9	13.79	1	iter2
NBL	ASIC4	5	11.26	2	iter2
NBL	CNGB1	6	10.21	1	iter2
NHL	CD79B	9	238.17	1	iter1
NHL	MS4A1	7	178.40	1	iter1
NHL	PTGDS	8	133.42	5	iter3
NHL	CCL19	6	125.45	3	iter1
NHL	TCL1A	6	98.92	1	iter2
NHL	CCL18	6	82.72	5	iter2
NHL	PLA2G2D	6	81.67	1	iter2
NHL	LTB	5	70.11	4	iter3
NHL	CD19	8	68.00	1	iter1
NHL	LRMP	8	61.20	5	iter3
NHL	POU2AF1	8	57.50	2	iter1
NHL	CD22	8	51.87	1	iter2
NHL	DTX1	6	44.68	2	iter1
NHL	SPIB	7	42.42	1	iter1
NHL	AIM2	6	39.32	2	iter2
NHL	BTK	5	36.96	5	iter3
NHL	FAIM3	8	33.74	2	iter3
NHL	TNFRSF13C	9	33.18	1	iter2
NHL	POU2F2	8	32.32	3	iter2
NHL	BLK	6	30.58	2	iter1
NHL	IRF4	8	29.91	4	iter1
NHL	FAM129C	8	29.33	1	iter2
NHL	PAX5	10	29.04	1	iter2
NHL	KIAA0226L	8	25.71	2	iter1
NHL	STAP1	6	25.54	3	iter2
NHL	GZMK	10	22.90	1	iter3
NHL	CLEC17A	9	21.27	1	iter1
NHL	FCRL3	9	19.99	1	iter1

NHL	TLR10	10	19.88	1	iter3
NHL	P2RX5	9	19.20	2	iter3
NHL	RGS13	10	17.75	1	iter1
NHL	IL21R	7	17.20	1	iter2
NHL	FCRL2	9	17.02	2	iter2
NHL	CLECL1	9	16.84	1	iter3
NHL	CD70	6	16.61	2	iter3
NHL	AICDA	10	15.14	1	iter1
NHL	MEF2B	10	12.61	1	iter2
NHL	TNFRSF13B	6	12.13	2	iter1
OS	MMP9	8	1010.67	2	iter2
OS	ALPL	10	408.71	2	iter2
OS	IBSP	10	369.24	1	iter1
OS	MXRA8	9	241.62	3	iter3
OS	IFITM5	10	210.23	1	iter1
OS	TMEM119	6	143.33	1	iter3
OS	SP7	10	93.79	1	iter1
OS	COL11A1	5	84.52	1	iter1
OS	PANX3	10	63.78	1	iter1
OS	PTH1R	9	52.73	2	iter1
OS	PHOSPHO1	8	50.88	2	iter1
OS	SCARF2	5	49.05	4	iter2
OS	COL11A2	5	30.00	1	iter1
OS	OMD	8	29.70	1	iter3
OS	DLX3	7	28.64	1	iter2
OS	CA3	10	27.61	1	iter1
OS	PRSS35	9	19.00	1	iter1
OS	CDH15	10	18.33	1	iter1
OS	INSC	5	16.55	1	iter1
OS	ITGA10	8	15.74	1	iter2
OS	HAPLN1	5	14.22	1	iter1
OS	MTRNR2L12	5	13.44	3	iter1
OS	KIF26A	9	12.31	3	iter2
OS	CHAD	8	12.12	1	iter2
OS	NDNF	7	10.97	3	iter2
OV	MSLN	10	247.36	3	iter2
OV	FOLR1	7	172.51	2	iter1
OV	SPON1	6	159.02	1	iter1
OV	KLK7	7	96.18	1	iter1
OV	KLK6	5	78.40	3	iter1
OV	SOX17	10	74.04	3	iter1
OV	ATP6V1B1	9	64.03	2	iter1
OV	SCGB2A1	10	63.64	2	iter1
OV	WT1	9	46.36	3	iter1
OV	KLK5	5	44.99	2	iter2
OV	C1orf186	8	33.15	5	iter1
OV	TNNT1	6	31.30	5	iter2
OV	KCNK15	10	30.76	1	iter1
OV	UPK3B	5	30.56	3	iter1
OV	SCGB1D2	5	29.98	3	iter1
OV	APOA1	8	29.47	2	iter1
OV	KLK8	10	24.40	2	iter3
OV	DAPL1	9	23.37	4	iter2
OV	DOK5	7	20.74	2	iter2
OV	FOXJ1	9	20.68	2	iter2
OV	CRABP1	9	19.03	2	iter3

OV	LPAR3	7	18.76	2	iter3
OV	KLHL14	6	15.18	2	iter1
OV	CLDN16	10	14.87	2	iter1
OV	ADRA2C	5	14.31	1	iter3
OV	FGF18	5	13.91	2	iter2
OV	LYPD6B	5	12.73	1	iter2
OV	MUC16	9	12.02	1	iter1
OV	AC234582.1	10	11.68	1	iter1
OV	PNOC	6	11.58	2	iter1
OV	CCNA1	5	11.08	1	iter3
OV	WNT10A	10	10.84	1	iter3
PAAD	TFF1	9	254.76	4	iter1
PAAD	SFRP2	9	208.46	4	iter2
PAAD	CTSE	9	208.01	3	iter1
PAAD	INS	9	187.92	1	iter1
PAAD	OLFM4	8	178.37	4	iter2
PAAD	SPINK1	6	156.98	4	iter1
PAAD	TFF2	9	91.66	2	iter1
PAAD	REG1A	7	72.09	2	iter1
PAAD	TCN1	10	60.93	2	iter2
PAAD	SST	7	40.20	3	iter1
PAAD	ANXA10	8	40.07	2	iter1
PAAD	VSIG2	5	36.42	5	iter3
PAAD	CLDN2	8	35.26	4	iter2
PAAD	CXCL5	6	29.08	2	iter1
PAAD	TTR	7	27.71	3	iter1
PAAD	C7	8	23.04	5	iter1
PAAD	GCG	9	18.05	1	iter1
PAAD	PRSS1	9	15.40	1	iter1
PAAD	MUC6	8	13.39	1	iter3
PAAD	MUC5AC	6	12.61	1	iter3
PAAD	DPCR1	9	11.98	1	iter3
PCPG	CHGA	10	12154.29	1	iter1
PCPG	CHGB	10	7288.39	3	iter1
PCPG	DBH	10	2274.52	2	iter1
PCPG	DLK1	10	1648.97	2	iter1
PCPG	SCG2	10	1354.67	1	iter1
PCPG	TH	10	823.43	2	iter1
PCPG	IGF2	6	708.70	3	iter2
PCPG	EEF1A2	10	521.11	2	iter3
PCPG	PENK	10	497.65	1	iter1
PCPG	CARTPT	10	465.85	1	iter1
PCPG	BEX1	5	381.05	5	iter2
PCPG	SCG5	9	324.36	1	iter3
PCPG	DDC	7	226.33	1	iter2
PCPG	ATP1A3	6	164.00	2	iter2
PCPG	FAIM2	5	160.22	2	iter1
PCPG	CPLX2	5	154.83	1	iter2
PCPG	CHRNA3	8	143.24	2	iter1
PCPG	PHOX2A	5	132.35	2	iter1
PCPG	SYP	9	127.88	2	iter3
PCPG	TAGLN3	10	113.95	2	iter2
PCPG	INSM1	6	99.53	1	iter2
PCPG	HMP19	6	98.29	2	iter1
PCPG	SCG3	10	94.80	3	iter3
PCPG	PTPRN2	8	88.86	3	iter3

PCPG	PTPRN	5	86.35	2	iter2
PCPG	SYT4	6	85.58	2	iter2
PCPG	VAT1L	7	81.41	2	iter2
PCPG	ARHGAP36	8	80.66	2	iter2
PCPG	SLC18A1	10	79.90	1	iter1
PCPG	PCSK2	6	77.69	2	iter1
PCPG	VWA5B2	5	72.99	1	iter2
PCPG	DUSP26	6	63.01	4	iter2
PCPG	DNER	6	62.22	3	iter3
PCPG	CDK5R2	7	61.24	2	iter3
PCPG	PHOX2B	7	60.23	2	iter1
PCPG	SYT1	7	56.15	2	iter2
PCPG	PNMT	5	52.98	1	iter2
PCPG	FAM163B	6	41.82	2	iter1
PCPG	DRD2	8	41.75	1	iter1
PCPG	SPOCK3	6	39.43	1	iter3
PCPG	TMEM179	5	36.27	2	iter2
PCPG	ATCAY	7	34.16	3	iter3
PCPG	C18orf42	6	30.34	1	iter1
PCPG	FEV	10	29.33	2	iter3
PCPG	PRLHR	8	22.41	1	iter1
PCPG	MAB21L1	9	21.65	2	iter2
PCPG	RAB3C	5	21.02	2	iter3
PCPG	SLC35D3	6	19.76	1	iter2
PCPG	DRGX	6	16.04	1	iter2
PCPG	FAM19A3	6	13.04	1	iter3
PCPG	SLC24A2	6	11.72	1	iter2
PCPG	DGKK	10	10.65	1	iter3
PCT	IGJ	10	8679.96	1	iter2
PCT	MZB1	5	870.58	1	iter2
PCT	FOSB	5	482.12	1	iter1
PCT	TRIB1	9	369.21	2	iter3
PCT	DERL3	6	304.89	1	iter1
PCT	DUSP5	7	300.03	1	iter2
PCT	TNFRSF17	10	288.04	1	iter2
PCT	SLAMF7	6	266.41	1	iter1
PCT	TXNDC5	10	164.08	1	iter1
PCT	IRF4	8	154.63	4	iter1
PCT	GPRC5D	10	137.29	1	iter1
PCT	BMP6	9	125.00	1	iter1
PCT	HLA-DOB	5	90.32	2	iter2
PCT	CPNE5	6	88.74	1	iter2
PCT	LAMP5	9	70.15	1	iter3
PCT	BHLHA15	5	63.25	1	iter1
PCT	FCRL5	9	53.01	1	iter2
PCT	ZBP1	6	42.64	1	iter1
PCT	FCRLA	9	41.23	3	iter1
PCT	P2RX1	5	40.97	4	iter3
PCT	GNG7	6	39.92	4	iter1
PCT	CH507-42P11.8	9	39.18	1	iter2
PCT	AMPD1	6	38.55	1	iter1
PCT	ICAM3	5	35.80	3	iter3
PCT	CCR2	5	31.84	1	iter3
PCT	ALDH1L2	6	27.86	1	iter3
PCT	MIXL1	7	20.57	1	iter2
PCT	DKK1	6	20.13	1	iter3

PCT	NDNF	9	17.07	3	iter2
PCT	FCRL2	7	16.62	2	iter2
PCT	LRRN1	10	16.25	3	iter3
PCT	UTS2R	9	16.04	1	iter1
PCT	CYSLTR1	5	11.53	1	iter3
PCT	DUSP26	6	11.45	4	iter2
PRAD	KLK3	10	4062.65	1	iter1
PRAD	MSMB	10	1736.91	1	iter1
PRAD	KLK2	10	970.11	1	iter1
PRAD	ACPP	10	894.93	1	iter1
PRAD	SLC45A3	7	482.24	1	iter1
PRAD	KLK4	10	413.51	1	iter1
PRAD	NPY	10	411.06	3	iter1
PRAD	AZGP1	6	399.74	3	iter1
PRAD	NKX3-1	10	385.52	1	iter1
PRAD	PLA2G2A	10	267.23	2	iter1
PRAD	PRAC1	10	245.55	1	iter1
PRAD	SLC44A4	5	211.18	5	iter2
PRAD	HOXB13	10	180.45	1	iter1
PRAD	OR51E2	10	171.28	1	iter1
PRAD	MYH11	10	136.61	1	iter2
PRAD	FOXA1	7	126.17	2	iter2
PRAD	FOLH1	10	110.95	1	iter2
PRAD	STEAP2	8	82.69	1	iter2
PRAD	ALDH1A3	7	71.63	1	iter3
PRAD	TRPM8	10	61.30	1	iter1
PRAD	ZG16B	6	42.00	3	iter3
PRAD	TMEFF2	6	35.73	1	iter1
PRAD	SLC4A4	7	35.57	5	iter3
PRAD	LRRC26	6	31.59	1	iter2
PRAD	ANO7	7	27.80	1	iter3
PRAD	UPK3A	10	27.53	2	iter2
PRAD	CHRM1	9	26.30	1	iter1
PRAD	MYBPC1	5	23.45	1	iter1
PRAD	PRAC2	10	23.36	1	iter2
PRAD	CHRNA2	10	22.76	1	iter1
PRAD	CPNE4	10	20.34	1	iter1
PRAD	ACSM1	8	19.44	1	iter2
PRAD	CWH43	10	17.01	2	iter2
PRAD	RAB3B	8	16.51	1	iter2
PRAD	MB	6	15.23	1	iter3
PRAD	TRPV6	10	14.62	1	iter2
PRAD	OR51E1	10	13.49	1	iter2
PRAD	ADRB1	6	11.43	1	iter2
PRAD	CUX2	6	10.21	1	iter3
SARC	MFAP5	5	20.46	1	iter3
SARC	TWIST1	6	18.71	3	iter2
SARC	DES	5	18.64	4	iter1
SARC	MRGPRF	7	16.22	2	iter2
SARC	TCEAL7	5	10.34	5	iter1
SKCM	PMEL	10	2006.33	2	iter1
SKCM	APOD	10	425.01	1	iter2
SKCM	S100B	8	376.70	2	iter1
SKCM	TYR	10	264.49	2	iter1
SKCM	MLANA	10	249.78	2	iter1
SKCM	SERPINE2	9	168.89	2	iter2

SKCM	PRAME	7	139.98	4	iter2
SKCM	TYRP1	9	100.77	2	iter1
SKCM	EDNRB	8	84.34	4	iter2
SKCM	DCT	10	67.44	1	iter1
SKCM	GPR143	6	64.73	2	iter1
SKCM	PLP1	10	64.34	2	iter1
SKCM	LZTS1	5	48.76	3	iter1
SKCM	BIRC7	9	40.97	3	iter2
SKCM	MITF	8	37.03	4	iter3
SKCM	SNCA	5	36.88	1	iter3
SKCM	SLC45A2	10	36.13	2	iter1
SKCM	MIA	7	36.02	1	iter1
SKCM	GYG2	8	34.67	3	iter2
SKCM	GPM6B	8	34.45	3	iter3
SKCM	MMP17	6	26.10	3	iter3
SKCM	TRIM63	6	20.60	2	iter1
SKCM	EXTL1	7	19.41	2	iter2
SKCM	FCRLA	8	19.29	3	iter1
SKCM	DAAM2	7	18.32	4	iter3
SKCM	CA14	5	16.05	2	iter1
SKCM	CRTAC1	6	15.83	3	iter2
SKCM	CORO2B	7	14.85	2	iter3
SKCM	BCHE	6	11.15	2	iter2
SKCM	GAPDHS	6	10.32	2	iter1
STAD	TFF1	10	153.21	4	iter1
STAD	SPINK1	10	117.18	4	iter1
STAD	CTSE	5	96.69	3	iter1
STAD	CLDN18	10	64.30	2	iter1
STAD	TMEM238	5	37.97	4	iter1
STAD	PGC	10	36.51	3	iter1
STAD	CST1	6	33.41	4	iter1
STAD	AGR3	6	29.98	5	iter2
STAD	REG4	9	29.26	3	iter1
STAD	TFF2	8	23.75	2	iter1
STAD	PHGR1	9	22.83	2	iter2
STAD	ACTG2	7	22.47	5	iter1
STAD	GATA6	5	22.00	5	iter2
STAD	HNF4A	5	18.23	4	iter2
STAD	DMBT1	6	17.15	3	iter3
STAD	MUC3A	7	15.91	2	iter3
STAD	GREM1	6	14.20	1	iter3
STAD	FOXA3	5	13.56	4	iter3
STAD	SOX18	6	12.93	4	iter3
STAD	GATA4	10	11.80	2	iter1
STAD	FOXA2	6	11.63	4	iter2
STAD	CXCL5	10	10.57	2	iter1
TGCT	POU5F1	7	365.40	1	iter1
TGCT	DPPA5	9	247.01	1	iter1
TGCT	KHDC3L	10	178.34	1	iter1
TGCT	L1TD1	8	110.09	1	iter1
TGCT	DPPA3	8	101.65	1	iter1
TGCT	CLDN6	7	94.81	1	iter1
TGCT	NLRP7	7	75.86	1	iter1
TGCT	GDF3	9	73.96	1	iter1
TGCT	LIN28A	9	64.25	1	iter1
TGCT	NANOG	8	53.33	1	iter1

TGCT	DPPA4	9	43.61	1	iter1
TGCT	UTF1	10	40.60	1	iter2
TGCT	ZFP42	5	37.67	1	iter1
TGCT	ALPPL2	8	35.32	1	iter2
TGCT	VENTX	7	28.22	1	iter3
TGCT	SLC7A3	5	28.10	1	iter1
TGCT	MT1H	5	25.08	1	iter3
TGCT	GIP	8	24.49	1	iter1
TGCT	TRIM71	10	22.27	1	iter2
TGCT	TRIML2	5	21.68	1	iter1
TGCT	RPRM	6	20.44	2	iter2
TGCT	PRDM14	8	19.38	1	iter1
TGCT	VRTN	8	14.60	1	iter1
TGCT	HDGFL1	10	14.11	1	iter2
TGCT	LIM2	10	12.85	1	iter2
TGCT	FOXH1	10	11.71	1	iter2
TGCT	NANOGP8	5	10.55	1	iter1
THCA	TG	10	2330.47	1	iter1
THCA	ZCCHC12	10	119.95	1	iter2
THCA	S100A1	7	108.00	4	iter1
THCA	PAX8	6	107.94	5	iter1
THCA	TSHR	10	95.03	1	iter1
THCA	NKX2-1	10	94.30	2	iter1
THCA	CITED1	9	92.11	4	iter1
THCA	SFTPB	8	87.15	4	iter1
THCA	KCNJ16	9	72.57	3	iter1
THCA	FOXE1	6	70.02	1	iter1
THCA	ZBED2	6	56.91	2	iter1
THCA	C2orf40	10	54.89	1	iter3
THCA	INPP5J	6	54.11	2	iter2
THCA	MUC15	6	51.19	1	iter1
THCA	IYD	10	48.93	1	iter1
THCA	SFTA3	7	48.54	2	iter1
THCA	LCN12	7	40.16	1	iter1
THCA	IGSF1	8	39.43	1	iter1
THCA	SNX22	10	39.20	1	iter2
THCA	LMO3	5	32.02	2	iter1
THCA	DCSTAMP	8	31.60	1	iter1
THCA	RXRG	5	30.52	2	iter2
THCA	PCSK2	6	30.31	2	iter1
THCA	KCNJ15	9	27.50	3	iter3
THCA	PDE8B	6	25.60	1	iter2
THCA	NTRK2	7	24.37	2	iter3
THCA	FCGBP	9	24.04	2	iter3
THCA	TPO	10	23.04	1	iter1
THCA	ATP13A4	8	21.44	1	iter3
THCA	PEBP4	5	18.85	2	iter3
THCA	SLC26A7	7	17.34	2	iter3
THCA	SLC26A4	9	17.00	1	iter2
THCA	TCERG1L	6	16.91	1	iter1
THCA	SLC30A2	7	15.88	1	iter2
THCA	SLC27A6	8	14.53	1	iter3
THCA	ST6GAL2	7	12.16	1	iter2
THCA	CRYGN	10	11.45	1	iter2
THCA	KLHDC8A	7	11.03	2	iter2
THYM	CCL25	10	1596.52	1	iter1

THYM	S100A14	5	512.46	5	iter2
THYM	CD3D	6	411.37	1	iter3
THYM	PRSS16	10	363.23	1	iter2
THYM	TBATA	10	341.35	1	iter1
THYM	CD1E	7	226.06	1	iter1
THYM	DNTT	10	218.85	2	iter1
THYM	CD1B	10	199.09	1	iter1
THYM	CD1A	5	151.55	1	iter1
THYM	PTCRA	6	148.57	1	iter1
THYM	PAX1	10	135.90	1	iter1
THYM	CD8B	5	125.21	1	iter3
THYM	CD1C	6	63.09	1	iter3
THYM	SH2D1A	5	62.64	1	iter3
THYM	CTSV	6	56.93	2	iter2
THYM	FAM107A	6	45.69	2	iter2
THYM	FOXN1	10	43.78	1	iter1
THYM	PSMB11	10	40.68	1	iter1
THYM	RAG1	9	39.39	1	iter1
THYM	CBFA2T3	5	32.25	4	iter2
THYM	KREMEN2	9	30.72	1	iter2
THYM	BEND5	6	28.44	2	iter3
THYM	SIX1	7	23.77	2	iter2
THYM	APOBEC2	7	22.92	1	iter2
THYM	PAX9	9	22.09	1	iter1
THYM	FAM163A	6	20.15	3	iter1
THYM	RAG2	9	18.49	1	iter1
THYM	PNMAL2	6	13.46	1	iter2
THYM	CCR9	8	12.87	1	iter2
UCEC	WFDC2	10	894.38	3	iter1
UCEC	SCGB2A1	10	281.58	2	iter1
UCEC	MSX1	10	100.97	1	iter1
UCEC	SOX17	10	100.22	3	iter1
UCEC	CAPS	7	65.70	2	iter1
UCEC	DLX5	10	48.34	2	iter1
UCEC	ASRGL1	7	42.41	4	iter2
UCEC	HOXB6	5	26.75	4	iter2
UCEC	VTCN1	7	26.18	4	iter1
UCEC	ESR1	10	21.47	2	iter1
UCEC	SCGB1D2	7	21.36	3	iter1
UCEC	MECOM	5	20.70	4	iter2
UCEC	HOXB8	9	18.72	3	iter2
UCEC	HOXB5	8	18.16	2	iter3
UCEC	LGR5	8	13.67	1	iter1
UCEC	C2orf88	6	11.02	2	iter3
UVM	PMEL	10	2725.92	2	iter1
UVM	TYRP1	10	1171.35	2	iter1
UVM	MLANA	10	654.15	2	iter1
UVM	CITED1	10	401.19	4	iter1
UVM	CRTAC1	9	268.31	3	iter2
UVM	EFHD1	9	213.95	3	iter1
UVM	RAB38	9	213.21	2	iter2
UVM	SLC7A8	5	212.90	1	iter2
UVM	TYR	10	182.13	2	iter1
UVM	MLPH	6	107.90	4	iter3
UVM	TUBB4A	5	101.47	5	iter2
UVM	LRRN4CL	5	91.27	1	iter1

UVM	BIRC7	7	77.52	3	iter2
UVM	SLC6A17	7	72.12	1	iter1
UVM	GPRC5B	7	61.09	4	iter3
UVM	GAPDHS	10	56.40	2	iter1
UVM	OCA2	5	56.16	1	iter1
UVM	MITF	7	55.51	4	iter3
UVM	LGI3	8	48.79	1	iter1
UVM	PAX3	10	44.69	2	iter1
UVM	D4S234E	7	42.99	4	iter2
UVM	CA14	9	40.37	2	iter1
UVM	KIT	9	37.94	4	iter3
UVM	SLC7A4	9	34.85	2	iter1
UVM	TRPM1	10	28.49	1	iter1
UVM	HSPB2	10	26.04	1	iter2
UVM	RASGRP3	10	25.11	3	iter2
UVM	RGS20	7	22.94	1	iter3
UVM	SLC45A2	10	22.86	2	iter1
UVM	ALX1	9	22.83	2	iter2
UVM	EXTL1	9	20.56	2	iter2
UVM	ABCB5	8	19.50	1	iter2
UVM	CAPN3	9	16.78	1	iter3
UVM	SOX1	7	16.22	1	iter2
UVM	PKNOX2	8	15.92	1	iter3
UVM	STK32A	5	15.81	1	iter3
UVM	ABCC11	9	13.18	1	iter1
UVM	TMEM200C	9	11.82	1	iter2
UVM	ASB11	10	10.25	1	iter2
